# Deciphering the molecular mechanism of the bacterial division motor TolQRA

**DOI:** 10.1038/s41421-025-00841-w

**Published:** 2025-11-04

**Authors:** Chongrong Shen, Teng Xie, Yongbo Luo, Fangyuan Zhao, Xin Wang, Zhibo Zhang, Jie Pang, Jierou Zhang, Xintan Dong, Shenghai Chang, Bi-Sen Ding, Binwu Ying, Wei Chi, Zhaoming Su, Ruhong Zhou, Xiaodi Tang, Haohao Dong

**Affiliations:** 1 Department of Laboratory Medicine, State Key Laboratory of Biotherapy, National Clinical Research Center for Geriatrics, West China Hospital, Sichuan University, Chengdu, China; 2 Shenzhen Eye Hospital, Shenzhen Eye Medical Center, Southern Medical University, Shenzhen, China; 3 Institute of Quantitative Biology, College of Life Sciences, Cancer Center, The First Affiliated Hospital, School of Medicine, Zhejiang University, Hangzhou, Zhejinag China; 4 Shanghai Institute for Advanced Study, Zhejiang University, Shanghai, China; 5 Department of Pathology of Sir Run Run Shaw Hospital, Zhejiang University School of Medicine, Hangzhou, Zhejiang China; 6 Department of Chemistry, Columbia University, New York, NY USA

**Keywords:** Cryoelectron microscopy, Cell division

## Abstract

The Tol-Pal system is essential for maintaining outer membrane (OM) stability during cell division in Gram-negative bacteria. The inner membrane complex TolQRA harnesses proton motive force (PMF) to establish transient interactions within the periplasm, thereby coordinating cell envelope remodeling and facilitating OM invagination at division sites. However, the precise mechanism remains unclear. Here, we present cryo-electron microscopy structures of *Escherichia coli* TolQRA in multiple conformational states at 2.92–3.52 Å resolution, revealing rotary dynamics within the complex. Computational simulations reveal a proton-conductive channel comprising the putative proton-accepting residue Asp23 and the conserved polar residues Thr145 and Thr178, with monitored inter-residue distances providing support for a proton-driven rotary mechanism. Site-directed mutagenesis combined with functional assays validates the AlphaFold-predicted structure of the periplasmic domains of TolR and TolA, and further pinpoints critical residues required for complex function. Together, these findings advance our understanding of TolQRA-mediated proton transduction and offer new avenues for antibiotic drug development.

## Introduction

The envelope of Gram-negative bacteria is characterized by an additional layer of the outer membrane (OM), which serves as a natural protective barrier against harmful compounds, including antibiotics^1–3^. OM lipoproteins like Braun’s lipoprotein (Lpp) and the peptidoglycan-associated lipoprotein (Pal) preserve the structural integrity of the OM by anchoring to the peptidoglycan (PG) layer^4–6^. During cellular division, the bacterial envelope undergoes significant morphological changes that involve PG remodeling and the contraction of the OM at the mid-cell^7–9^. This complex process requires precise coordination among the three envelope compartments to uphold the structural and functional integrity of the resulting daughter cells^10,11^. In the late stage of bacterial division, the Tol-Pal system plays a crucial role in synchronizing the invagination and contraction of the OM with the separation of the newly synthesized septal PG layer^12–14^. Deficiencies in this system destabilize the OM and increase bacterial susceptibility to antimicrobial agents, making the Tol-Pal system a promising target for developing novel antibacterial therapies^15–20^.

**Fig. 1:**
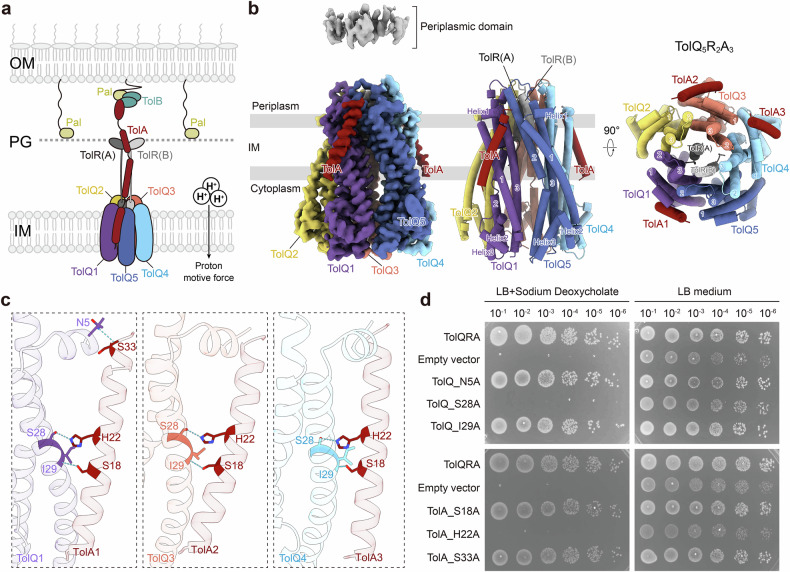
Cryo-EM structure of TolQRA. **a** Schematic diagram of the Tol-Pal system. The Tol complex transduces PMF to synchronize dynamics in cell envelope compartments. TolB sequesters Pal in the OM for anchoring to the PG layer. Upon association with the Tol system, Pal becomes available for interaction with PG. **b** Cryo-EM map and structure of the TolQRA complex from the side and top views, showing the IM motor unit and periplasmic domains. TolQ1(purple), TolQ2 (yellow), TolQ3 (orange), TolQ4 (light blue), TolQ5 (blue), TolA (red), TolR chain A (gray) and TolR chain B (light gray). **c** Interactions between TolQ and TolA, specifically, TolQ1–TolA1, TolQ3–TolA2, and TolQ4–TolA3 from left to right. The amino acids forming hydrogen bonds are highlighted. **d** Viability assays of site-directed mutagenesis of the interacting residues shown in **c**.

The Tol-Pal system consists of five core components: TolQ, TolR, TolA, TolB, and the Pal protein. It functions through coordinated interactions among components distributed across different compartments of the bacterial envelope^21–23^ (Fig. 1a). TolQ and TolR form a transmembrane complex in the inner membrane (IM), which harnesses the proton motive force (PMF) to mediate energy transduction and establish connectivity between the IM and the OM^24^. Structural studies of the TolQR complex reveal structural homology to the bacterial flagellar stator complex MotAB^25,26^ and the TonB-dependent nutrient transporter complex ExbBD^27,28^, both of which are prokaryotic rotary motors that transduce PMF energy to induce conformational changes in downstream effector components^29^. It has been proposed that TolQ induces conformational changes in the periplasmic domains of TolR and TolA^30^. Structural analyses through X-ray scattering and crystallography reveal that TolA comprises a short N-terminal transmembrane segment (domain I), followed by an elongated, stalk-like domain (domain II), and a C-terminal globular domain (domain III)^31^. Biochemical analyses indicate that TolA domain III (TolA_III) is crucial for interacting with the soluble protein TolB^12^ (Supplementary Fig. S1a). Free TolB is thought to bind to OM-anchored Pal. Upon binding to TolA-III, TolB dissociates from Pal, allowing Pal to engage with the PG layer at the division site, thereby facilitating localized OM constriction for division^12,32,33^ (Fig. 1a).

The TolQRA knockout strain fails to undergo proper cell division and displays a characteristic multi-segmented, chain-like morphology under low osmotic conditions^13,14^. This phenotype can be suppressed by overexpression of soluble PG hydrolase^13^, highlighting the role of TolQRA in coordinating OM invagination and PG remodeling. However, the mechanism by which TolQRA mediates transient tethering between the cell wall and the OM remains unclear^34^. In this study, we captured cryo-electron microscopy (cryo-EM) structures of the TolQRA complex in multiple conformational states. Structural comparisons allowed us to investigate the rotary motion of TolQR driven by PMF. Combined with molecular dynamics (MD) simulations of the proton conduction pathway within TolQR and functional analysis of the periplasmic domains of TolRA, we proposed a model for proton-induced mechanical transduction by the Tol-Pal system. Additionally, key residues crucial for the function of TolQ, TolR, and TolA were identified through cell viability and morphological analysis. These findings provide critical insights that could inform the development of novel antimicrobial strategies.

## Results

### The overall architecture of the TolQRA complex

Previous live fluorescent microscopy results suggest that TolA is dynamically recruited to the cell septum via TolQR^35,36^. However, the assembly of the complex has not been directly visualized. To address this, we co-expressed TolQR and TolA from *Escherichia coli* (strain K12), purified, and determined the structure of the TolQRA complex reconstituted in phospholipid nanodiscs by cryo-EM (Supplementary Figs. S1b, S2 and Table S1). The transmembrane portion of the complex, including TolQ and the transmembrane helices of TolR and TolA, is well resolved at 3.52 Å resolution. Densities for the periplasmic domains of TolA and TolR were obtained at lower resolution due to inherent flexibility (Fig. 1b and Supplementary Fig. S2). These regions were subsequently modeled through AlphaFold3 prediction^37^. TolQ forms a pentameric structure similar to that of MotA and ExbB, exhibiting a volcano-like configuration with a narrow periplasmic end and a wider cytoplasmic base (Fig. 1b). The pentameric TolQ encapsulates the dimeric TolR through its N-terminal transmembrane helices at the periplasmic end. Each TolQ monomer comprises three transmembrane helices (TM1–TM3). Adjacent monomers interact through their TM2 and TM3 helices to assemble a hollow central core composed of ten TM helices, while TM1 is positioned peripherally, interfacing with the TM2–TM3 junction (Fig. 1b). The periplasmic ends of the central pore exhibit a slight clockwise twist when viewed from the periplasm, while the cytoplasmic ends remain largely perpendicular to the membrane plane. The N-terminus of TM1 extends into a short periplasmic helix (helix 1) that lies parallel to the membrane, and the linker between TM1 and TM2 forms two cytoplasmic helices (helix 2 and helix 3) oriented obliquely at the outer base of the central core (Fig. 1b).

In the TolQRA nanodisc complex, we identified three major reconstructions showing up to three TolA molecules associated with TolQ subunits TolQ1, TolQ3, and TolQ4, respectively (Fig. 1b and Supplementary Fig. S2). TolA interacts with TolQ through their transmembrane helices (TolA_I) in a crossing configuration, stabilized by hydrogen bonds between TolQ_I29 and TolA_S18, TolQ_S28 and TolA_H22, and TolQ_N5 and TolA_S33 (Fig. 1c). These interactions are consistent with the conserved SHLS motif shared by TolA and its homolog TonB^38,39^. MD simulations revealed that the hydrogen bonds between TolQ_I29 and TolA_S18 and TolQ_S28 and TolA_H22 are stable (Supplementary Fig. S1d). Site-directed mutagenesis disrupting the TolA_H22A-TolQ_S28A interaction impaired bacterial viability in the presence of the membrane-permeable agent sodium deoxycholate (DOC) (Fig. 1d), indicating that this interaction is vital for proper Tol complex function. Despite this, the variant proteins can still be co-purified as a complex (Supplementary Fig. S1c), suggesting that TolA and TolQR interact through additional interfaces, likely involving the periplasmic domain of TolR (Fig. 1b).

### Mapping the proton channel within the deprotonated TolQR complex

**Fig. 2:**
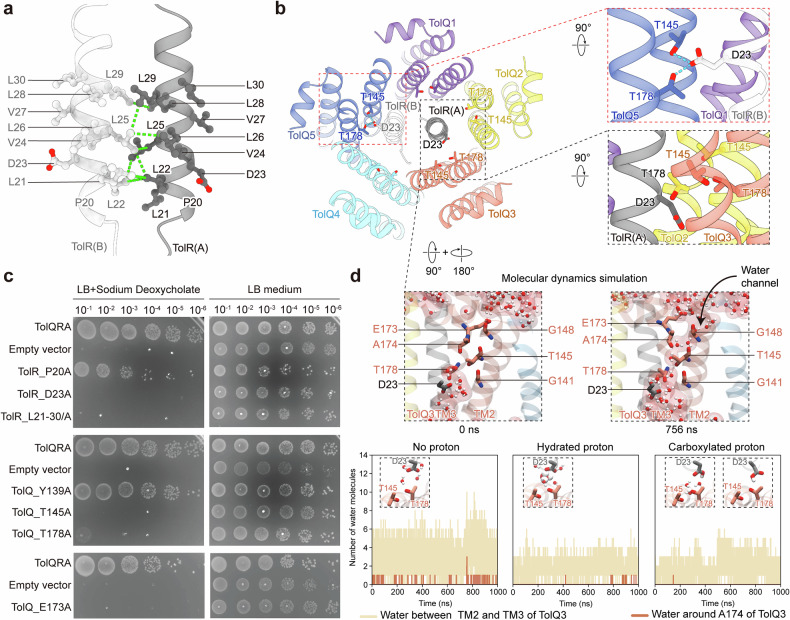
Identification of the proton channel in TolQ. **a** The interactive interface between the transmembrane helix of TolR dimer, showing a leucine-rich motif (L21LDVLLVLLL30). The side chains of amino acids L21–L30 are depicted, and the interactions are highlighted. **b** Top view of the TolQR structure, showing the channel cavity. The interactions between the putative proton acceptor D23 of TolR and the conserved T145 and T178 of TolQ are highlighted (right boxed panel). The side chain of D23 on TolR chain B is oriented towards TolQ5 (blue), forming hydrogen bonds with TolQ_T145 and TolQ_T178. The side chain of D23 on TolR chain A is oriented towards TolQ2 (yellow) and TolQ3 (orange) and shows no interaction with the conserved threonines T145 and T178 (right boxed panel). **c** Cell viability of mutants with substitutions in the TolR N-terminus and the putative proton channel of TolQ. **d** MD simulations reveal the formation of a water channel between TM2 and TM3 of the TolQ subunit 3, with channel-related side chains depicted. Simulations of water entry into the channel under different protonation states are shown (D23, being deprotonated, hydrated, and protonated states from left to right).

The pentameric TolQ structure exhibits a hydrophobic region near the periplasmic end, delineating the transmembrane region, while the remaining surface is predominantly hydrophilic (Supplementary Fig. S3a). The internal cavities correspond to these hydrophobic and hydrophilic regions. TolR engages with TolQ within the hydrophobic cavity through the TolR transmembrane helices (TolR_TMs) (Supplementary Fig. S3a). The TolR_TMs dimerize through a leucine-rich motif (L21LDVLLVLLL30) (Fig. 2a). Substitution of these leucine residues with alanine resulted in cell death in the presence of DOC (Fig. 2c), highlighting their functional importance. Notably, the conserved charged residue D23 within this motif is shared among homologous prokaryotic motor proteins and is proposed to play a role in proton binding and transfer^40,41^. The asymmetric architecture of TolQ leads to an uneven spatial arrangement of its pentameric subunits relative to the central TolR axis. While D23 from TolR chain A (TolR(A)_D23) points toward the interface between TolQ2 and TolQ3, TolR(B)_D23 forms hydrogen bonds with T145 and T178 of TolQ5 (Fig. 2b and Supplementary Fig. S3b). T145 and T178 from the TolQ pentamer form a polar ring within the hydrophobic transmembrane cavity. This striking structural feature, conserved in both TolQ and ExbB^40,41^, likely facilitates proton conduction into the otherwise nonpolar environment. Site-directed mutagenesis of TolR_D23A, TolQ_T145A, and TolQ_T178A resulted in bacterial death in the presence of DOC and exhibited filamentous phenotypes, indicating that these residues are essential for function (Fig. 2c and Supplementary Fig. S3c, d). In MotAB, protonation of MotB_D22 in chain A and chain B alternately stabilizes its interaction with T189 in each corresponding MotA subunit, with each proton uptake event driving a 36° stepwise rotation of the complex^25,26,42^. Similarly, our TolQRA structure reveals an analogous asymmetry: TolR_D23 in chain A and chain B adopt distinct conformations relative to TolQ, positioning them for protonation-dependent rearrangements reminiscent of the MotAB mechanism.

To investigate the proton transport path within the TolQRA complex, we conducted MD simulations around the putative proton acceptor TolR_D23 (Fig. 2d and Supplementary Fig. S3e). The threonine-interacting TolR(B)_D23 was fixed in the protonated state, and TolR(A)_D23 was simulated in either protonated or unprotonated forms. During microsecond simulations, a single-file water chain was observed from the periplasmic side between the TM2 and TM3 helices of TolQ3, approaching the unprotonated TolR(A)_D23 (Fig. 2d and Supplementary Fig. S3e). This linear water chain likely mediates proton translocation via a Grotthuss-like mechanism^43^. In contrast, under the simulated protonation state of TolR(A)_D23, water occupancy around TM2 and TM3 of TolQ3 was markedly reduced. These results suggest that the electronegativity of TolR(A)_D23 is important for the formation of a proton-conductive pathway. Along the water channel, we also identified a polar residue TolQ_E173 at the periplasmic entrance of the channel (Fig. 2d). Its negatively charged side chain projects into the periplasm, potentially serving as a proton attractor for its translocation. Substitution of E173 with alanine (TolQ_E173A) caused bacterial lethality and disrupted cell division, highlighting the functional significance of this residue (Fig. 2c and Supplementary Fig. S3d).

### Rotary motion of the TolQR motor unit

**Fig. 3:**
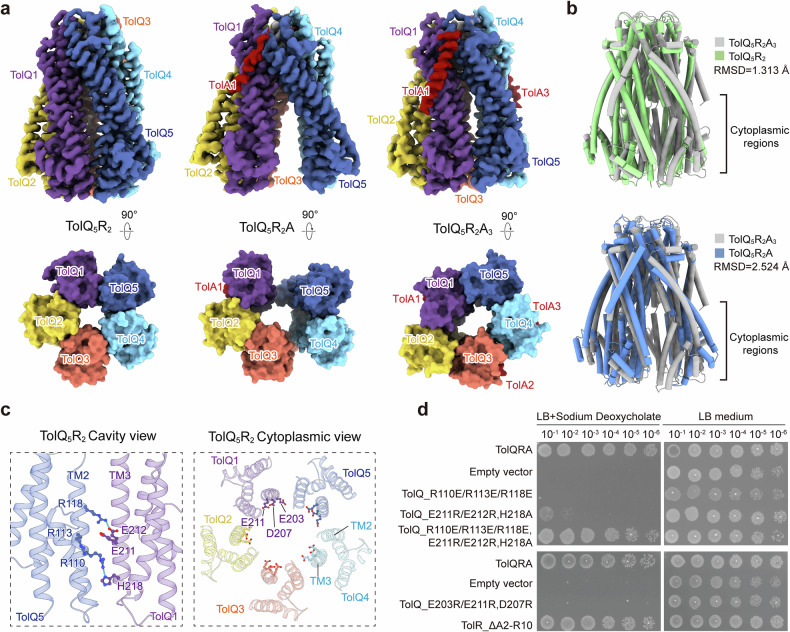
The cryo-EM structure of TolQRA in distinct conformations. **a** The cryo-EM maps of the SMA-reconstituted structures of TolQ_5_R_2_, TolQ_5_R_2_A, and nanodisc-reconstituted TolQ_5_R_2_A_3_ from the side and cytoplasmic views. The color scheme is the same as in Fig. 1. **b** Structural superimposition of the cryo-EM structures of TolQ_5_R_2_ (grass green), TolQ_5_R_2_A (sky blue) and TolQ_5_R_2_A_3_ (gray) from the side view, using TolR dimer as a superimposed reference. **c** Interactions between TolQ subunits near the cytoplasmic end (represented using the TolQ_5_R_2_ structure), showing salt bridge formation between the positively charged residues in TM2 and negatively charged residues in TM3 (left panel). A row of negatively charged residues faces the cytoplasmic cavity (right panel). **d** Cell viability assays of the interactive residue variants shown in **c**.

To explore the dynamic motion of the TolQRA complex, we aimed to capture its distinct states using styrene-maleic acid (SMA) polymers. SMA enables the extraction of membrane proteins directly from their native lipid bilayer, preserving the complex in its biological state^44^. Two distinct structural classes with high resolutions were determined at 2.92 and 3.28 Å, respectively, with one class lacking density for TolA (hereafter TolQ_5_R_2_) and the other displaying density corresponding to one copy of TolA (hereafter TolQ_5_R_2_A) (Fig. 3a and Supplementary Figs. S4, S5a, and Table S1). The TolA molecule bound in TolQ_5_R_2_A is positioned consistently with its location on the TolQ1 subunit in the nanodisc TolQ_5_R_2_A_3_ structure. The superimposition of the TolQ_5_R_2_ and TolQ_5_R_2_A structures onto the TolQ_5_R_2_A_3_ structure shows root-mean-square deviation (RMSD) values of 1.31 and 2.52 Å, respectively, based on the alignment of 217 Cα atoms (Fig. 3b and Supplementary Fig. S5b).

The superimposition of these structures, utilizing the TolR dimer as a reference, revealed that the TolQ_5_R_2_ structure slightly rotated compared to TolQ_5_R_2_A_3_. The morphing transition between TolQ_5_R_2_A_3_ and TolQ_5_R_2_ demonstrates a prominent shutter-like rotation in the cytoplasmic termini of TolQ in relation to the central TolR dimer (Supplementary Videos S1, S2). The prominent conformational changes at the cytoplasmic ends of the motor unit indicate high dynamics in these regions. When viewed from the cytoplasmic side of the pentamer, TolQ_5_R_2_A_3_ features an oval-shaped cytoplasmic opening, TolQ_5_R_2_ presents a round opening, and TolQ_5_R_2_A displays a split between TolQ1 and TolQ5 as well as between TolQ3 and TolQ4 (Fig. 3a). Conformational variation primarily arises from the cytoplasmic half of the TM2 helix (Supplementary Fig. S6a), which contains three arginine residues (R110, R113, and R118). These residues form salt bridges with glutamic acid residues (E211 or E212) and hydrogen bonds with H218 in TM3 of adjacent TolQ subunits (Fig. 3c and Supplementary Fig. S6a, b). Substitution of these arginines with glutamic acid (R110E/R113E/R118E) disrupted the interactions and impaired bacterial viability (Fig. 3d and Supplementary Fig. S6c). Interestingly, introducing compensatory charge-reversal mutations in TM3 (E211R/E212R) restored viability when combined with the TM2 arginine substitutions (R110E/R113E/R118E) (Fig. 3d), indicating that electrostatic interactions between TM2 and TM3 are essential for TolQ function, likely by stabilizing the channel architecture and preventing proton leakage. Along the cytoplasmic end of TM3, additional negatively charged residues, E203 and D207, extend their side chains toward the channel lumen without forming direct interactions (Fig. 3c). Mutational analysis revealed that substitution of these residues (E203R, D207R, and E211R) significantly impaired bacterial viability, highlighting their functional importance (Fig. 3d and Supplementary Fig. S6c). In the homologous MotA protein, the acidic cytoplasmic region has been proposed either to serve as a proton reservoir or to mediate interactions with the N-terminus of MotB^42^. Our result supports the former hypothesis, as deletion of the TolR N-terminus (TolRΔ2–10) had no detectable effect on cell viability (Fig. 3d and Supplementary Fig. S6c).

To investigate how D23 protonation in TolR influences conformational dynamics during TolQRA rotation, we performed MD simulations on different protonation states of the SMA-captured intermediate TolQ_5_R_2_ structure (Supplementary Fig. S7b). Protonation of D23 in both TolR(A) and TolR(B) enhanced structural stability, as reflected by consistent TolR_D23–TolQ_T178 distances in the simulations (Supplementary Fig. S7b). These results suggest that D23 protonation promotes conformational stabilization following a rotation step initiated by proton transduction at the previously deprotonated D23 site. Notably, stabilization of the TolR_D23–TolQ_T178 interaction correlated with a more consistent positioning of the resolved N-terminal region of TolR(A) (residues 14–17) relative to TolQ1–5 (Supplementary Fig. S7a, b), implying a structural link between D23 protonation and TolQ rotational motion. Further structural analysis revealed that TolR residues S14, I16, and N17 form contacts with TolQ residues G131 and Y139 (Supplementary Fig. S7a). Substitution of Y139 with alanine did not affect cell viability (Fig. 2c), whereas replacing it with a charged residue was lethal (Supplementary Fig. S5c), indicating that the hydrophobic character of Y139 is functionally essential, possibly to prevent proton leakage during transduction. Together, these findings, supported by MD simulations and structural morphing analysis, suggest a rotary mechanism analogous to that of the MotAB complex, but with distinct features of proton translocation.

### TolA interacts with the periplasmic domain of TolR at the dimer interface

**Fig. 4:**
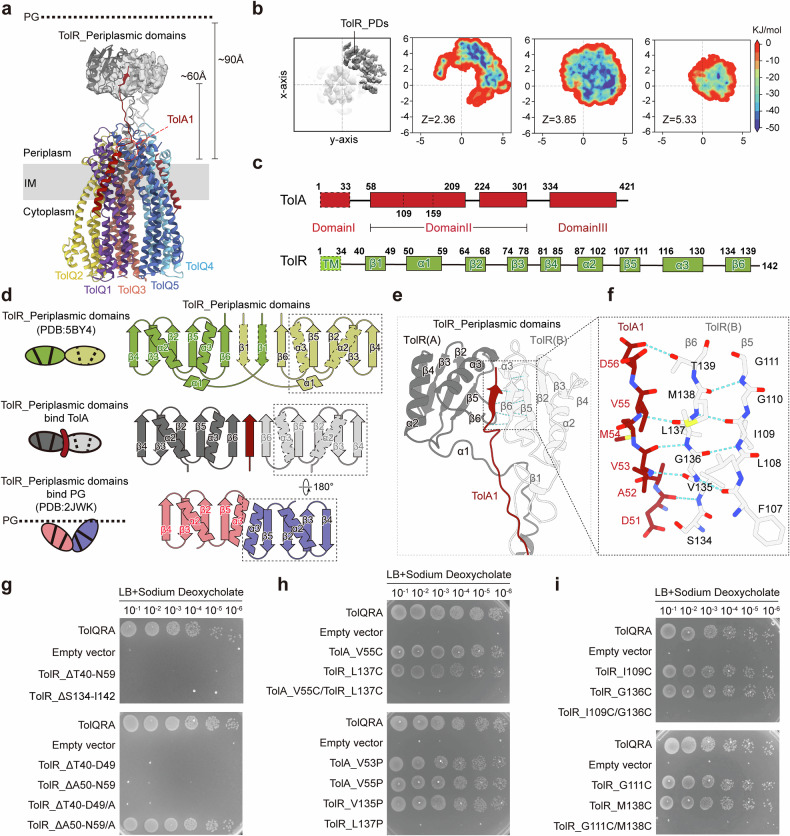
Characterization of the periplasmic domains of TolQ_5_R_2_A_3_. **a** A remote map density was obtained in the cryo-EM structure of TolQ_5_R_2_A_3_, 60 Å above the IM motor unit. **b** Conformational sampling of the TolA–TolR periplasmic domains by metadynamics. Free-energy surface reconstructed from multiple-walker well-tempered metadynamics simulations, projected onto the relative center-of-mass displacement (X, Y, Z) between the TolR_PDs-TolA1_loop and the TolR transmembrane reference. The results reveal that TolR is confined to a restricted region of conformational space, corresponding to less than a full 360° rotational range, when engaged with TolA. **c** Schematic diagram showing subdomains of TolA and TolR. **d** Topological diagram of the TolR dimer, showing remodeling of its conformations. The inactivated conformation (top, green/yellow) with the dimeric interface of β6/β1/β1/β6 (crystal structure, PDB: 5BY4). The TolA interaction conformation (mid, gray/red), showing the interface β6/TolA/β6 (our structure). The activated conformation (bottom, pink/purple), showing the interface of β5/β5 (crystal structure, PDB: 2JWK). **e** The TolR periplasmic region interacted with the periplasmic loop of TolA, which was modeled using AlphaFold3. **f** Details of the interactive interface among TolA_loop, TolR_β6 and TolR_β5 with hydrogen bond indicated. **g** Cell viability assays of TolR mutants with truncated subdomains indicated in **c**. **h**, **i** Cell viability assay of crosslinking mutants between TolA_β and TolR_β6 (**h**) and TolR_β6 and TolR_β5 (**i**).

In the cryo-EM map, we also observed a satellite periplasmic density located ~60 Å above TolQ5 and TolQ1, which likely corresponds to the periplasmic domains of TolR (TolR_PDs) (Fig. 4a). Previously reported crystal structures of the isolated TolR_PDs from *E. coli* (PDB: 5BY4) and *H. influenzae* (PDB: 2JWK) showed two distinct conformations: one with a flat interface and the other with a bent interface (Supplementary Fig. S8a). It has been proposed that the TolR_PD dimer undergoes “strand-swapping” remodeling upon activation, in which one monomer rotates 180°, converting the dimer from C2 symmetry to an asymmetrical architecture (Supplementary Fig. S8b). This remodeled interface has been experimentally shown to mediate PG binding and resembles the dimeric structures of other PG-binding proteins such as Pal, MotB, and ExbD^4,45–48^. Although the resolution of the periplasmic density is relatively low, its flat shape and the sub-PG height imply that these TolR_PDs are in their pre-remodeled conformation (Fig. 4a and Supplementary Fig. S8c). MD simulations of the dimeric TolR_PDs reveal a restricted range of motion, with rotational flexibility limited to less than 360° (Fig. 4b). This motion is confined near TolQ1 and TolQ5, indicating that the TolR_PDs are anchored at that location, likely due to the interactions with the periplasmic loop connecting domains I and II of TolA1. A segment of this TolA1_loop is resolved in the cryo-EM map, protruding into the periplasmic space from the interface between helices 1 of TolQ1 and TolQ5, aligning with the TolR_PDs (Fig. 4a). Consequently, we predicted TolR_PDs-TolA1_loop structure using AlphaFold3, which revealed structural features resembling the crystal structure of the pre-remodeled TolR_PD dimer (PDB: 5BY4), supporting our interpretation of the observed periplasmic density (Fig. 4a).

Each monomer of the TolR_PDs contains six β-strands (β1–β6) and three α-helices (α1–α3) (Fig. 4c, d). Studies of homologous proteins suggest that strands β1 and β6 are involved in TolR dimerization, and the remodeling of the TolR_PDs is achieved through rearrangement of the dimerization interface from β6/β1’/β1/β6’ to β5/β5’^45^ (Fig. 4d). In our structure, the TolR_PD dimer exhibits C2 symmetry (Fig. 4d and Supplementary Fig. S8a). The N-terminal β1 and α1 from both TolR_PD monomers are shown as unfolded loops (Fig. 4e). The remaining strands β2–β6 form β-sheets, and the two α-helices (α2, α3) are positioned on the same side (Fig. 4d, e). While β6 of TolR chain A is disordered, β6 of TolR chain B interacts with the periplasmic TolA_loop through a β-strand (residues 52–55, TolA_β hereafter) at the dimeric interface (Fig. 4e, f). Functional assays showed that deletion of β6 (TolR^Δ134–142^) or β1–α1 (TolR^Δ40–59^) of TolR resulted in cell death in the presence of DOC (Fig. 4c, g). Furthermore, the TolR^Δ134–142^ disrupted the complex assembly between TolA and TolQR, highlighting the importance of TolR_β6 in maintaining the interaction between TolA and TolR. Deleting either β1 (TolR^Δ40–49^) or α1 (TolR^Δ50–59^) of TolR independently was also lethal (Fig. 4g and Supplementary Fig. S9a, b). Interestingly, replacing the deleted β1 or α1 with an equally long alanine linker restored the lethality effects caused by TolR^Δ50–59^ but not by TolR^Δ40–49^ (Fig. 4g and Supplementary Fig. S9a, b). This suggests that TolR_α1 likely functions as a linker, but TolR_β1 plays a role that requires residue specificity.

At the dimeric interface, TolR_β6 interacts with TolA_β through backbone hydrogen bonds between TolR_L137 and TolA_V55 and between TolR_V135 and TolA_V53 (Fig. 4f). Cysteine mutations at these residues crosslinked the complex to a higher molecular weight (Supplementary Fig. S9b), validating the predicted interface between TolR_β6 and TolA_β. Preventing hydrogen bond formation at TolR_L137 by substituting it with the kinky proline (TolR_L137P) resulted in cell death in the presence of DOC (Fig. 4h and Supplementary Fig. S9a, b), suggesting that interactions between TolR_β6 and TolA_β1 are critical for mediating the transduction role of the Tol system. Moreover, crosslinking of this site also caused lethality (Fig. 4h and Supplementary Fig. S9a, b), suggesting that the interaction needs to be transient for proper functionality.

### Remodeling of the periplasmic domain of the motor unit

In the predicted structure, the periplasmic loop of TolA adopts a stretched configuration, while the loop of TolR is compressed (Fig. 4e). This suggests that the TolR_PDs could potentially extend further, but in the current conformation, their extension is restricted by the length of the TolA loop. In the crystal structure of activated TolR_PDs (PDB: 2JWK), β6 of TolQ_PDs is absent from the dimeric interface (Supplementary Fig. S8a). We speculate that rotation of the motor unit drives the elevation of the TolR_PDs toward the PG layer, and this upward movement facilitates interface remodeling, as the tethered TolR_β6 strand is pulled away from the dimer interface by TolA_β. Supporting this model, covalent crosslinking β6 to β5 at the TolR interface led to bacterial cell death in the presence of DOC, indicating that dissociation of TolR_β6 from the interface is essential for function (Fig. 4i and Supplementary Fig. S9a, b). This remodeling likely results in the formation of a new β5/β5 interface and a grooved, eight-stranded β-sheet that mediates PG binding. Notably, TolR lacking both β1/α1 and β6 (TolR^60–133^) retained the affinity with TolA regardless of the presence of the periplasmic loop TolA_β (Supplementary Fig. S9c). This implies that the remodeled β5/β5 TolR_PD dimer also associates with TolA but through a region other than TolA_β. This observation is consistent with the idea that the remodeled TolR_PD dimer, upon associating with the PG layer, may provide mechanical support to the rigid helical domain II of TolA, enabling it to traverse the PG layer and engage TolB. Supporting this notion, a previous study demonstrated that replacing TolA_II with a flexible loop impaired its function^30^.

## Discussion

The Tol-Pal system is essential for maintaining the integrity of the bacterial cell envelope and facilitating OM constriction during cell division. The TolQR complex acts as a motor/stator unit that establishes a connection between the inner and outer membranes using a PMF transducing mechanism that is not fully understood. In this study, we determined the cryo-EM structures of the TolQRA complex in distinct states. Combined with MD simulations and cellular functional analyses, our findings reveal molecular details suggesting a PMF-driven rotary mechanism in TolQR and spatial coupling of TolR and TolA through their periplasmic domains.

The overall structure of the TolQR complex shares homology with the ExbBD complex (Supplementary Fig. S10a), implying a conserved mechanism of the PMF transducers. A recent study developed a chimeric ExbBD–TonB^TM^-TolA complex that retrieved the lethal effects of the *TolQRA* knockout strain^49^, highlighting the interchangeable roles of these PMF transductors in regulating TolA. Interestingly, a recent study on the *Acinetobacter baumannii* TolQR complex revealed a distinct TolQR conformation, in which the TolR helices adopt translational symmetry, unlike the rotary symmetry observed in ExbBD and in our *E. coli* TolQRA structure. The work proposed a mechanism in which conformational changes in TolR reposition the putative proton acceptor D27 toward the TolQ pore, thereby unplugging the channel to permit ion passage^50^. In contrast, our *E. coli* TolQRA structures align more closely with the architecture reported by Hervé et al.^51^ (Supplementary Fig. S10b), supporting a conserved mechanism shared between TolQR and ExbBD. Further structural analysis and MD simulations allowed us to model a rotary mechanism that, while conserved, exhibits distinct features compared to the previously proposed MotAB mechanism.

Using MD simulations, we identified for the first time the emergence of a single-file water chain within the TolQRA complex, positioned between the transmembrane helices TM2 and TM3 of a TolQ unit (TolQ3). This linear pathway, which likely facilitates proton translocation via a Grotthuss-like mechanism, begins at the periplasmic residue TolQ3_E173, traverses a polar ring formed by T145 and T178, and terminates at the putative proton acceptor TolR(A)_D23. Water influx into the hydrophobic cavity occurred only when TolR(A)_D23 was unprotonated (Fig. 2d and Supplementary Fig. S3e), suggesting that the negative charge facilitates the formation of a proton-conductive channel. In contrast, no water channel was detected near the protonated TolR(B)_D23, which interacts with TolQ_T145/T178 in TolQ5. This interaction may neutralize the polar pocket within the hydrophobic cavity, thereby preventing hydrated proton access. Interestingly, the location of the proposed proton channel differs between the MotAB and TolQR complexes. In MotAB, the channel lies at the interface between two adjacent MotA units, whereas in TolQR, it is confined within a single TolQ unit between TM2 and TM3 (Fig. 2d). This architectural distinction likely reflects underlying differences in channel formation: in TolQ, key residues T145 and T178 within a single subunit form the channel, while in MotAB, polar residues T155 and T189 from TM3 and TM4 of neighboring MotA units contribute to the channel structure^25^.

Upon protonation, TolR(A)_D23 is expected to engage the T145/T178 site of TolQ3, potentially triggering a rotary power stroke within the motor complex. The selective formation of the proton-conductive channel specifically in TolQ3, but not in TolQ2, suggests a directional rotary mechanism. As TolQ rotates, TolR(B)_D23 disengages from the threonine pair, and the released proton is likely drawn into the acidic cytoplasmic cavity. Supporting this, charge-altering mutations within the cytoplasmic cavity are lethal (Fig. 4c, d), underscoring the functional significance of its electronegativity in the Tol-Pal system. Following deprotonation, TolR(B)_D23 reorients to accept a new proton, initiating the next step in the power stroke cycle.

The 5:2 stoichiometry of the TolQR complex presents a conserved structural framework across diverse bacterial energy-transducing systems, such as PomA₅B₂, ZorA₅B₂ and MotA_5_B_2_. While this architecture is essential for coupling ion translocation to mechanical output, the downstream functions of these complexes diverge. In MotA₅B₂ and PomA₅B₂, the stator–rotor interaction directly produces torque to drive flagellar rotation, powered by proton or sodium gradients, respectively^52^. ZorA₅B₂ is thought to recruit cytoplasmic effectors to initiate signal transduction in response to envelope stress^53^. In contrast, the TolQ₅R₂ complex neither generates torque nor transmits cytoplasmic signals. Instead, it harnesses the PMF to activate the periplasmic effector TolA, promoting OM constriction during cell division. Cryo-EM structures reveal assemblies of TolQR associated with varying numbers of TolA (ranging from one to three) (Figs. 1b, 3a). Despite the presence of multiple TolA molecules, structural and functional analyses suggest that only a single TolA is modulated by TolQR at any given time to exert its function (Fig. 4e–i). The periplasmic domain of the TolR dimer (TolR_PD) aligns with the domain II loop of TolA (TolA_II loop), and their interaction interface with their corresponding β-strands (TolR_β6/TolA_β) was predicted based on conserved sites identified in the related Ton system^48^. The TolR_β6/TolA_β interface was experimentally validated by the formation of disulfide bonds upon double cysteine substitutions (Fig. 4h and Supplementary Fig. S9b). Interestingly, both disruption of hydrogen bonding and covalent crosslinking between TolR_β6 and TolA_β abolished bacterial growth (Fig. 4h), suggesting that this interaction is only required transiently. TolR_β6 is proposed to regulate the conformational state of the TolR_PD dimer, transitioning its interface from β6/β6 (inactive) to β5/β5 (active) (Fig. 4d). During the rotary motion of the motor unit, TolA_β may exert a pulling force on TolR_β6, disengaging it from the dimeric interface and facilitating TolR_PD activation.

**Fig. 5:**
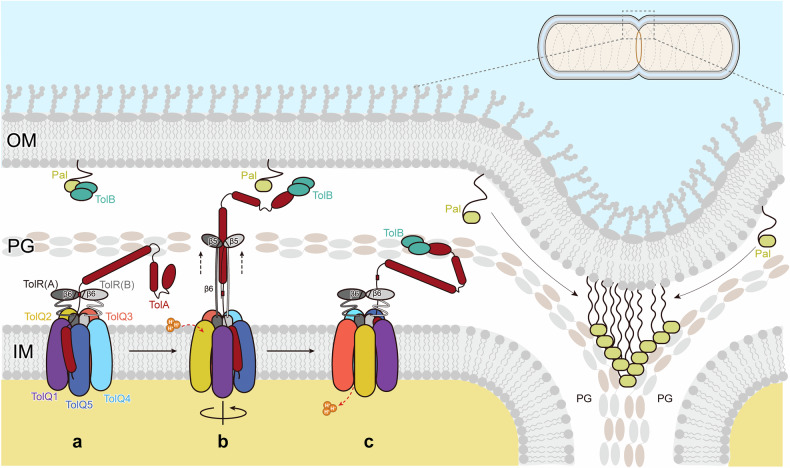
Mechanism of TolQRA operation diagram. **a** TolA associates with the TolQ complex, where its periplasmic loop interacts with the periplasmic interface of TolR via the β6 strand. **b** Upon proton transfer, the PMF drives the rotation of the motor unit, which swings the periplasmic domain of TolR. Due to its tethering to TolA, this swinging force promotes the disengagement of β6 from the TolR periplasmic interface, remodeling its conformation to the activated state that is capable of coupling with the PG layer. The extended TolR periplasmic dimer provides a supporting force to the domain II helix of TolA, which holds it to traverse the PG layer to fish for TolB. **c** Further rotation brings TolA away, breaking the interaction between β6 and TolA_loop, causing the complex to return to its relaxed conformation.

The activated TolR_PD dimer is reported to interact with the PG layer^45^, implying that its activation is accompanied by an elevation in height following the rotary stroke. In our structure of TolQRA, the inactive TolR_PD dimer is positioned approximately 60 Å above the membrane plane (Figs. 4a, 5a), which is below the height of the PG layer^54^, reinforcing the hypothesis that activation involves a structural elevation. Functional studies suggest that a manually activated TolR_PD dimer (deleting β1 and β6) retained affinity for TolA_II lacking the β-sheet-containing loop (Δβ) (Supplementary Fig. S9c). This finding implies that PG-bound TolR_PD may interact with TolA_II at an alternative site, likely along the long helix of TolA_II (Fig. 5b). The interaction between this rigid region of TolA and PG-bound TolR_PD could generate a holding force, enabling the remaining portions of TolA_II and TolA_III to extend into the upper periplasmic space, where they facilitate the recruitment of TolB from the OM (Fig. 5c).

Our study provides key insights into the mechanistic operation of the Tol-Pal system. Structural and functional analyses support a proton-driven rotary mechanism in which TolQR modulates the dynamics of TolA and TolR, facilitating critical interactions within the periplasmic space. These findings highlight the transient yet essential nature of TolR–TolA interaction and suggest that disrupting this coordination impairs bacterial growth. Given the essential role in maintaining OM stability, the Tol-Pal system presents a promising target for antimicrobial development, where interfering with the TolQR-driven mechanical cycle or TolA-TolR coupling could compromise bacterial viability.

## Methods and materials

### Overexpression and purification of TolQRA complex

The operons encoding TolQRA from the *E. coli* K12 strain were cloned into the pTRC99a plasmid at the *Xba*I and *Sal*I restriction sites, resulting in the pTRC99a-TolQRA construct. Subsequently, a strep-tag and an eight-histidine (8× His) tag were introduced at the C-termini of TolR and TolA, respectively.

The resultant pTRC99a-TolQR_Strep_A_His_ plasmid was transformed into *E. coli* C43 (DE3) cells (Novagen) for protein expression. The transformed cells were cultured at 37 °C in LB medium supplemented with 100 µg/mL ampicillin. Protein expression was induced by adding 0.2 mM IPTG once the culture reached an optical density of 0.8 at 600 nm (OD_600_), and the incubation continued for 3 h at 37 °C. Cells were harvested by centrifugation and resuspended in lysis buffer A (20 mM Tris-HCl, pH 7.8, 300 mM NaCl, 10% (vol/vol) glycerol) supplemented with 0.1 mM PMSF. The cells were then lysed, and the membranes were collected by ultracentrifugation at 140,000× *g* for 1 h at 4 °C. Membrane proteins were extracted in buffer A supplemented with 1% (wt/vol) n-dodecyl-β-d-maltopyranoside (DDM; Anatrace) and 0.1% (wt/vol) cholesteryl hemisuccinate (CHS; Anatrace) for 1 h at room temperature. After ultracentrifugation at 140,000 × *g* for 1 h, the solubilized protein suspension was applied to a HisTrap HP column (5 mL, GE Healthcare) for purification. The column was washed with buffer A containing 30 mM imidazole, 0.02% (wt/vol) lauryl maltose neopentyl glycol (LMNG; Anatrace), and 0.004% CHS, and the bound protein was eluted with buffer A containing 250 mM imidazole, 0.02% LMNG and 0.004% CHS. The eluted protein was further purified using a Superdex 200 Increase 10/300 column (GE Healthcare) equilibrated with buffer B (20 mM Tris-HCl, pH 7.8, 150 mM NaCl) supplemented with 0.02% LMNG and 0.004% CHS. The purified fractions of the TolQRA complex were concentrated for nanodiscs reconstitution.

### Reconstitution of TolQRA in lipid-nanodiscs

The purified TolQRA complex was reconstituted into lipid nanodiscs using membrane scaffold protein MSP1D1 and *E. coli* total lipid extract (Avanti Polar Lipids). Lipids were dissolved in chloroform and subsequently dried under a gentle stream of nitrogen to form lipid films, which were then placed under vacuum overnight to remove residual solvent. The dried film was rehydrated, briefly sonicated, and homogenized to a concentration of 10 mg/mL in buffer B (20 mM Tris, pH 7.8, 150 mM NaCl) supplemented with 200 mM sodium cholate. The purified TolQRA protein, MSP1D1 membrane scaffold protein, and lipids were combined at a molar ratio of 1:2:200 in buffer B. The mixture was incubated for 1 h at 4 °C to allow nanodisc assembly, followed by the addition of 300 mg Bio-Beads SM2 (Bio-Rad) to initiate detergent removal. After 4 h incubation at 4 °C with gentle mixing, the Bio-Beads were removed, and the nanodisc-reconstituted TolQRA was further purified using a Superdex 200 column equilibrated with buffer B. The TolQRA nanodisc fractions were pooled and concentrated using a 100-kDa cutoff Amicon Ultra-15 centrifugal filter (Merck-Millipore) in preparation for cryo-EM analysis.

### Purification of TolQRA in SMA-nanodiscs

Cells transformed with the TolQRA plasmid were cultured as previously described. Cell pellets were collected by centrifugation and resuspended in lysis buffer A (20 mM Tris-HCl, pH 7.8, 300 mM NaCl, 10% (vol/vol) glycerol) containing 0.1 mM PMSF. The cells were disrupted, and cell debris was removed by centrifugation (5000 × *g*, 20 min, 4 °C). The lysate was then subjected to ultracentrifugation (140,000 × *g*, 1 h, 4 °C) to pellet the membranes. The membrane pellets were homogenized in 30 mL of SMA-nanodisc buffer (2.5% SMALP 200 (Cube Biotech), 20 mM Tris-HCl, 300 mM NaCl, 10% glycerol, 0.1 mM PMSF, pH 7.8) and incubated at room temperature for 5 h with constant inversion. The solution was ultracentrifuged (140,000 × *g*, 30 min, 4 °C), and the supernatant was collected and loaded onto a StrepTrap HP column (5 mL, GE Healthcare) for purification. The column was washed with buffer C (20 mM Tris, pH 7.8, 500 mM NaCl), and the bound protein was eluted with buffer B (20 mM Tris, pH 7.8, 150 mM NaCl) supplemented with 5 mM d-desthiobiotin. The native nanodisc constituted TolQRA was further purified using a Superdex 200 column equilibrated with buffer B. The protein fractions were concentrated for cryo-sample preparation.

### *E.coli TolQRA* knockout strain

To generate targeted chromosomal modifications, a kanamycin resistance cassette was amplified from plasmid pKD13 using primers with homology arms specific to the TolQ and TolA genomic regions^55–57^. *E. coli* strain MG1655 was first transformed with plasmid PKD46, which enables expression of λ-Red recombinase functions. Following induction of λ-Red expression for 1 h, the PCR amplified kanamycin resistance cassette was electroporated into electrocompetent MG1655 cells. Transformants were selected on LB agar plates supplemented with kanamycin and incubated at 37 °C. Positive recombinants were verified by PCR and confirmed by Sanger sequencing. To eliminate the temperature-sensitive pKD46 plasmid, verified strains were cultured at 37 °C for three successive passages before long-term storage.

### Site mutagenesis and cell viability assays

To enable detection by western blotting, a Myc tag was inserted between amino acids 224 and 225 of TolQ. Plasmids used for functional studies were generated by site-directed mutagenesis from the pTRC99a-TolQ_Myc_R_Strep_A_His_ construct.

The *TolQRA* knockout strain exhibits sensitivity to DOC, and 1% DOC was used as the selective pressure. Under these conditions, the *TolQRA* knockout strain is unable to grow; however, expression of plasmid TolQRA rescues growth, enabling functional complementation assays. For spot dilution assays, transformed *TolQRA* knockout cells were plated on LB agar plates supplemented with 100 µg/mL ampicillin and 50 µg/mL kanamycin and incubated at 37 °C for 15 h. A single colony from each transformation was inoculated into 5 mL of LB medium containing the same antibiotics and grown at 37 °C for 10 h. The cell pellets were washed with fresh LB, normalized to an OD_600_ of 1.0, and subjected to serial tenfold dilutions. Aliquots (4 µL) from dilutions ranging from 10^−1^ to 10^−6^ were spotted onto LB agar plates with or without 1% DOC, and incubated at 37 °C for ~15 h before imaging.

For expression analysis, the TolQRA in the knockout strain was expressed in 100 mL of LB medium using the same conditions as previously described above. Cells were harvested, resuspended in 4 mL of buffer A containing PMSF, and sonicated. DDM was added to a final concentration of 1% to solubilize membranes at room temperature for 30 min. The supernatant was loaded onto a 0.5 mL Ni-NTA agarose column (QIAGEN) and incubated for 40 min. The column was washed with buffer A containing 30 mM imidazole, 0.02% LMNG, and 0.004% CHS. Bound proteins were eluted with buffer A supplemented with 250 mM imidazole, 0.02% LMNG, and 0.004% CHS. Eluted proteins were analyzed for sodium dodecyl sulfate-polyacrylamide gel electrophoresis followed by western blot. Immunodetection was carried out using anti-His (Abcam, cat. ab18184), anti-Myc (Sigma, cat. A5963), and anti-Strep (Abcam, cat. ab184224) primary antibodies at a 1:3000 dilution. Protein bands were visualized by chemiluminescence using appropriate horseradish peroxidase (HRP)-conjugated secondary antibodies and imaged with a Bio-Rad imaging system.

### Microscopic observation of bacterial morphology

To evaluate the effects of TolQRA mutations on bacterial division, plasmids encoding either wild-type or mutant TolQRA were transformed into the *TolQRA* knockout strain. A single colony from each transformation was inoculated into LB medium containing the appropriate antibiotics and cultured at 37 °C for 10 h. The overnight cultures were adjusted to an OD_600_ of 1.0, then diluted 1:100 into low-salt LB medium supplemented with IPTG. Cultures were incubated at 30 °C for 2 h, followed by a temperature shift to 42 °C for 3 h to induce protein expression. Live cells were imaged at room temperature using a phase contrast microscope (Sunny Optical Technology).

### Microscale thermophoresis (MST) analysis of TolA-TolR binding

Gene fragments encoding TolR (residues 36–142 and 60–133) and TolA (residues 34–421 and 58–421) were amplified from *E. coli* K12 genomic DNA and individually cloned into a modified pET28a plasmid. For protein purification, a Strep-tag was fused to the C-terminus of TolR, while an octa-histidine tag (8× His) was added to the C-terminus of TolA. The resulting plasmids were transformed into *E. coli* BL21(DE3) for protein overexpression. Transformed cells were cultured in LB medium containing 50 μg/mL kanamycin at 37 °C until the culture reached an OD_600_ of 1.0. Protein expression was induced with 0.5 mM IPTG at 20 °C for 16 h. His-tagged soluble TolA in the lysate supernatant was purified using a Ni-NTA column and eluted with Buffer C (20 mM HEPES pH 7.8, 150 mM NaCl) containing 300 mM imidazole. Excess imidazole was removed by desalting using a HiPrep 26/10 column (GE Healthcare). Similarly, strep-tagged soluble TolR was purified via a Strep-Tactin column and eluted with Buffer C containing 50 mM biotin, followed by desalting to remove residual biotin.

For MST analysis, TolR (residues 36–142 or 60–133) were used as a ligand and concentrated to 300 µM. TolA (residues 34–421 or 58–421) was diluted to 200 nM in Buffer C, respectively. The RED-tris-NTA dye (MO-L018, NanoTemper Technologies) was diluted to 100 nM in the same buffer and mixed with TolA in a 1:1 volume ratio. The labeling reaction was in the dark at room temperature for 30 min. Prior to measurements, samples were centrifuged at 10,000 × *g*, for 10 min at 4 °C to remove aggregates.

For all MST experiments, the final concentration of labeled TolA was fixed at 50 nM. A 16-point half-log serial dilution of TolR ligands (ranging from 150 μM to 4.58 nM) was prepared. Each reaction consisted of 10 μL labeled TolA and 10 μL ligand solution. MST measurements were performed using a Monolith NT.115 instrument (NanoTemper Technologies) in standard capillaries at 25 °C. Data were analyzed using MO. Affinity software (v2.3) and results were further processed and visualized using GraphPad Prism 9.5.

### Cryo-EM sample preparation and data acquisition

The TolQRA nanodiscs (3 µL) and TolQRA SMA (3 µL) samples, both at a concentration of ∼4 mg/mL, were applied to glow-discharged Quantifoil grids (Cu, 1.2/1.3 µm hole size/spacing, 300 mesh) for 60 s. The grids were blotted for 2 s at 100% humidity and 4 °C, then plunge-frozen in liquid ethane cooled by liquid nitrogen using a Vitrobot Mark IV (Thermo Fisher Scientific).

Cryo-EM data were acquired on a Titan Krios microscope (Thermo Fisher Scientific) operated at 300 kV, equipped with a Quantum GIF energy filter (Gatan) with a 20 eV energy slit. Movies were recorded in counting mode on a Gatan K2 Summit direct electron detector at a nominal magnification of 130,000, corresponding to a pixel size of 1.1 Å. Defocus values ranged from –1.7 to –1.1 µm. Each stack was exposed for 7 s (TolQRA nanodisc) and 6 s (TolQRA SMA), and dose-fractionated into 35 frames (TolQRA nanodisc) and 30 frames (TolQRA SMA), with total electron doses of 57.63 e^−^/Å² and 45.66 e^−^/Å², respectively. Movie stacks were automatically collected using EPU software.

### Cryo-EM image processing

Image processing workflows for both datasets are summarized in Supplementary Figs. S2 and S4. All data processing was carried out using a combination of RELION v3.1^58^, cryoSPARC v4.4, and EMAN2.31.

For the nanodisc-reconstituted TolQRA dataset (Supplementary Fig. S2), 10,802 dose-fractionated movies underwent motion-correction using MotionCor2^59^, and contrast transfer function (CTF) parameters were estimated using CTFFIND4^60^. After discarding low-quality micrographs, 10,314 were retained for particle picking using EMAN2.31^61^, yielding 2,675,942 particles. Reference-free 2D classification was performed in cryoSPARC^62^, and 2,189,486 particles were selected for ab-initio reconstruction and six-class heterogeneous refinement. Among them, the first and third classes exhibited well-defined features of membrane proteins. Further low-pass filtering and heterogeneous refinement of these 2,189,486 particles yielded a class displaying the expected architecture. A total of 579,624 particles representing this pentameric TolQRA complex were selected for further 3D reconstruction and downstream analysis. In parallel, a separate class of 847,945 particles, corresponding to a hexameric TolQRA complex (six TolQ subunits), was identified and subjected to refinement only, to obtain a higher-resolution map but not analyzed further. A subset of 579,624 particles was selected for six-class ab initio reconstruction and heterogeneous refinement. From the first class, a well-resolved density of the periplasmic region of TolR was observed. A total of 181,516 particles were refined under C1 symmetry using homogeneous refinement, followed by map enhancement with DeepEMhancer. This resulted in a map with well-defined density for the flexible periplasmic domain of TolR (connected via a flexible linker), along with clearly resolved transmembrane helices of the TolQRA complex. Reference-based motion-correction refined 181,301 particles, which were further subjected to homogeneous refinement to yield the final density map featuring the periplasmic TolR region. Following this, 3D variability analysis was performed on the 181,301 particles. Six major clusters were identified, which primarily differed in the number of TolA subunits bound to the periplasmic region. Specifically: Cluster_1, Cluster_4, and Cluster_5 represented complexes with one TolA molecule bound; local refinement of their combined particles resulted in Conformation III, resolved at 3.93 Å from 93,628 particles; Cluster_0 and Cluster_2 represented two TolA-bound states, yielding Conformation II at 3.37 Å from 66,693 particles; Cluster_3 reflected binding of three TolA subunits, resulting in Conformation I at 3.41 Å from 21,195 particles. These three conformational states reveal dynamic association of TolA with the periplasmic region of the TolQRA complex.

For the SMA-constituted TolQRA dataset (Supplementary Fig. S4), 6618 dose-fractionated movies were motion-corrected using MotionCor2, and CTF estimation was performed using CTFFIND4. After quality filtering, 6554 micrographs were used for particle picking with EMAN2.31, generating 5,691,235 particles. After 2D classification in cryoSPARC, 3,889,018 particles were selected for ab-initio reconstruction and six-class heterogeneous refinement. Notably, the first and second classes exhibited distinct conformations in the transmembrane region. To further characterize these conformations, low-pass filtered reference maps were generated and used for heterogeneous refinement of 2,910,427 particles, yielding two conformational classes: Conformation I (the TolQR structure) and Conformation II (the TolQRA structure).

For Conformation I, 1,047,913 particles were selected and refined using a low-pass-filtered map as the initial model for two rounds of heterogeneous refinement, yielding a dominant class containing 84.3% of particles. A total of 646,331 particles from this class underwent homogeneous, non-uniform, and local refinement under C1 symmetry, producing a 3.05 Å resolution map. After reference-based motion-correction, 629,965 particles were further refined, resulting in the final density map of Conformation I.

For Conformation II, 716,620 particles were selected and similarly refined through two rounds of heterogeneous refinement, resulting in a split-density map showing bound TolA helical density (72.2%, 279,284 particles). To optimize this region, 3D variability analysis was performed, yielding six clusters. Four clusters (234,097 particles) showed well-defined TolA helical density and were further refined under C1 symmetry using homogeneous, non-uniform, and local refinement, generating a 3.40 Å resolution map. Reference-based motion-correction refined 228,227 particles, which were then subjected to local refinement, yielding the final Conformation II map with improved TolA density.

All final cryo-EM maps were sharpened using a negative B-factor automatically determined in cryoSPARC using a Guinier plot. Map resolution was evaluated based on the gold-standard Fourier shell correlation at a 0.143 threshold, and the local resolution was estimated within cryoSPARC. All refinements were performed under C1 symmetry.

### Model building and refinement

The AlphaFold3-predicted model served as an initial reference. The model was docked into the electron density using UCSF Chimera. Then, each individual residue was manually examined and adjusted to fit the map in Coot^63^. Subsequently, the model was refined against the corresponding map in PHENIX^64^. MolProbity was used to evaluate the stereochemistry and geometry of the structure^65^. TolQ_5_R_2_A_3_, TolQ_5_R_2_A, and TolQ_5_R_2_ were modeled into the cryo-EM map. All structure figures were prepared with PyMOL and UCSF ChimeraX^66^.

### MD simulations

Only the region of the TolQR structure that fitted into the cryo-EM maps was included in the MD simulations. All systems were constructed using the CHARMM-GUI membrane builder^67,68^. A proton was added to the residue Asp23 of one or both TolR subunits. The structures of the TolQR complex with different protonation states were embedded in a phospholipid bilayer composed of PE:PG (75:25). All systems were solvated with TIP3P water molecules. A 0.15 M KCl solution was added to neutralize the system. The final system size was approximately 170,000 atoms.

Simulations were performed using GROMACS (version 2022.5)^69^, with the CHARMM36m force field applied to proteins and CHARMM36 used for lipids^70^. Long-range electrostatic interactions were calculated using the particle mesh Ewald (PME) method^71^. A 12-Å cutoff was applied for Lennard-Jones interactions, with a switching function starting at 10 Å. The temperature was maintained at 310 K using the V-rescale thermostat with a coupling constant of 1 ps^72^, while the pressure was kept at 1 bar using the Parrinello–Rahman barostat^73^, which had a coupling constant of 5 ps. All hydrogen-containing covalent bonds were constrained using the LINCS algorithm^74^, enabling an integration time step of 2 fs. Before production ran, the system was energy-minimized, heated to 310 K, and pre-equilibrated in the canonical ensemble with position restraints applied to heavy atoms. Production simulations were then conducted for at least 1000 ns. Structural stability and hydration were evaluated by calculating RMSD, inter-residue distances, hydrogen bond counts, and water occupancy using VMD (version 1.9.3)^75^. To explore the conformational dynamics of the TolA–TolR periplasmic domains, well-tempered metadynamics^76^ combined with the multiple-walkers technique^77^ (MW-WT-MetaD) were performed using PLUMED (version 2.9)^78^^,79^ patched with GROMACS (version 2022.5). The MDFF-refined structure of the TolQ–TolR–TolA complex (TolQ, residues 5–221; TolR, residues 14–142; TolA, residues 14–56, with missing residues modeled) served as the starting configuration. Collective variables (CVs) were defined as the relative displacement of the center of mass (COM) of the TolR periplasmic domain and TolA loop (TolR, residues 63–142; TolA, residues 48–56) with respect to the TolR transmembrane reference motif (residues 31–33). The three Cartesian components of this COM displacement (X, Y, Z) were used as CVs. To restrict sampling to physically relevant states of the TolQ–TolR–TolA complex, Gaussian hills were deposited every 1 ps with an initial height of 0.3 kJ/moL and widths (σ) of 0.15 nm for each CV. A bias factor of 5 was employed in the well-tempered scheme, corresponding to an effective bias temperature of 1550 K, while the physical simulation temperature was maintained at 310 K. Six walkers were initialized from distinct regions of the CV space identified during preliminary sampling, with bias potentials exchanged every 0.2 ps. Simulations were performed for an aggregate sampling time of 1.2 μs. Free-energy surfaces were reconstructed from the accumulated bias using the sum_hills utility implemented in PLUMED.

## Supplementary information

Supplementary Materials

PDBs

cytoplasmic view of morphed conformational change

periplasmic view of morphed conformational change

## Data Availability

The cryo-EM density maps and the atomic model coordinates have been deposited at the Electron Microscopy Data Bank (www.ebi.ac.uk/pdbe/emdb) and the Protein Data Bank (www.rcsb.org), respectively, under the accession numbers EMDB: EMD-53394 and PDB: 9QVD (TolQRA in nanodisc), EMDB: EMD-70088 and PDB: 9O40 (TolQR in SMA), EMDB: EMD-53380 and PDB: 9QUQ (TolQRA in SMA).
