# Supplementary material for: Deciphering the molecular mechanism of the bacterial division motor TolQRA
Source: Cell Discov. 2025 Nov 4;11:87. doi: 10.1038/s41421-025-00841-w (PMC12583513; doi:10.1038/s41421-025-00841-w)
Supplement: Supplementary file 1 — Supplementary Materials [file 41421_2025_841_MOESM1_ESM.pdf]

Supplementary Materials for

Deciphering the molecular mechanism of the bacterial division motor TolQRA

Chongrong Shen[1,2]*, Teng Xie[3,4]*, Yongbo Luo[1]*, Fangyuan Zhao[1]*, Xin Wang[1]*, Zhibo Zhang[1], Jie Pang[1], Jierou Zhang[1], Xintan Dong[1], Shenghai Chang[5], Bi-Sen Ding[1], Binwu Ying[1], Wei Chi[2], Zhaoming Su[1#], Ruhong Zhou[3,4,6#], Xiaodi Tang[1#], Haohao Dong[1,2#]

[1]Department of Laboratory Medicine, State Key Laboratory of Biotherapy, National Clinical Research Center for Geriatrics, West China Hospital, Sichuan University, Chengdu, China.

[2]Shenzhen Eye Hospital, Shenzhen Eye Medical Center, Southern Medical University, Shenzhen, China.

[3]Institute of Quantitative Biology, College of Life Sciences, Cancer Center, The First Affiliated Hospital, School of Medicine, Zhejiang University, Hangzhou, China.

[4]Shanghai Institute for Advanced Study, Zhejiang University, Shanghai, China.

[5]Department of Pathology of Sir Run Run Shaw Hospital, Zhejiang University School of Medicine, Hangzhou, China.

[6]Department of Chemistry, Columbia University, New York, NY, USA.

*These authors contributed equally to this work.

#Correspondence: haohaodong@scu.edu.cn (H.D.), tangxiaodi@scu.edu.cn (X.T.), rhzhou@zju.edu.cn (R.Z.), zsu@wchscu.cn (Z.S.).

This material contains:

Supplementary Figs. S1-10 and Table S1

Supplementary video 1: Periplasmic view of morphed conformational change.mp4

Supplementary video 2: Cytoplasmic view of morphed conformational change.mp4

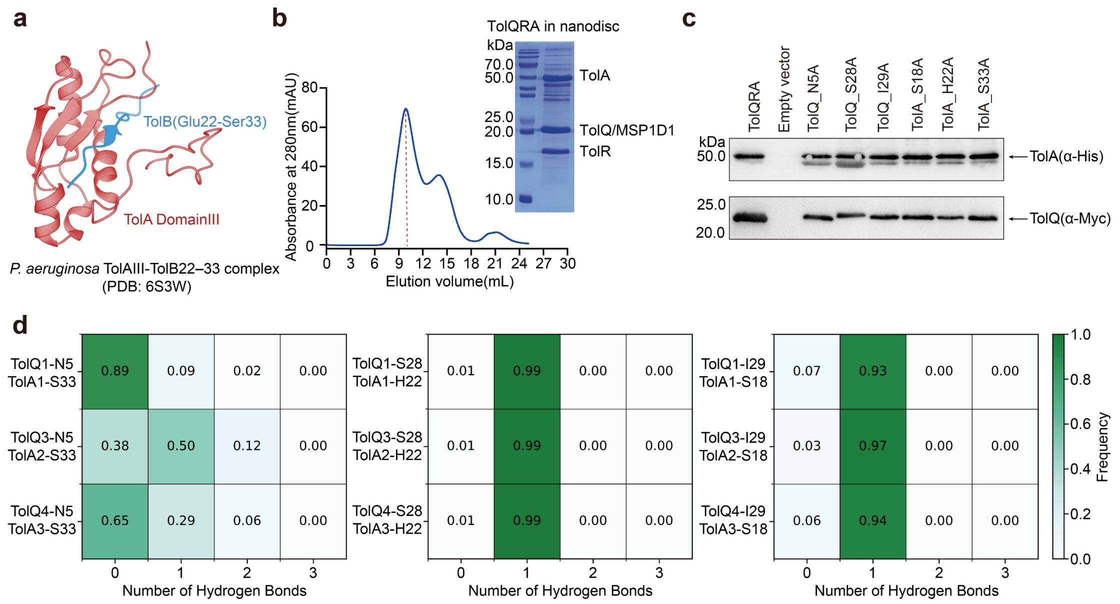

Fig. 1 | Analysis of TolA and TolQ interaction sites

(a) The crystal structure of TolA domain III that bound to TolB via segment Glu22-Ser33 (PDB: 6S3W). TolA is colored dark red and TolB is colored blue.

(b) SDS-PAGE and SEC profile of purified TolQRA reconstituted in nanodiscs. SDS-PAGE samples are eluted at the volume indicated by red dotted lines.

(c) Western blot detection of wild-type and mutant TolQRA shown in Figure 1d. Data are representative of n = 3 independent experiments.

(d) Molecular dynamics simulation of the number of hydrogen bonds formed by TolQ and TolA in Figure 1c.

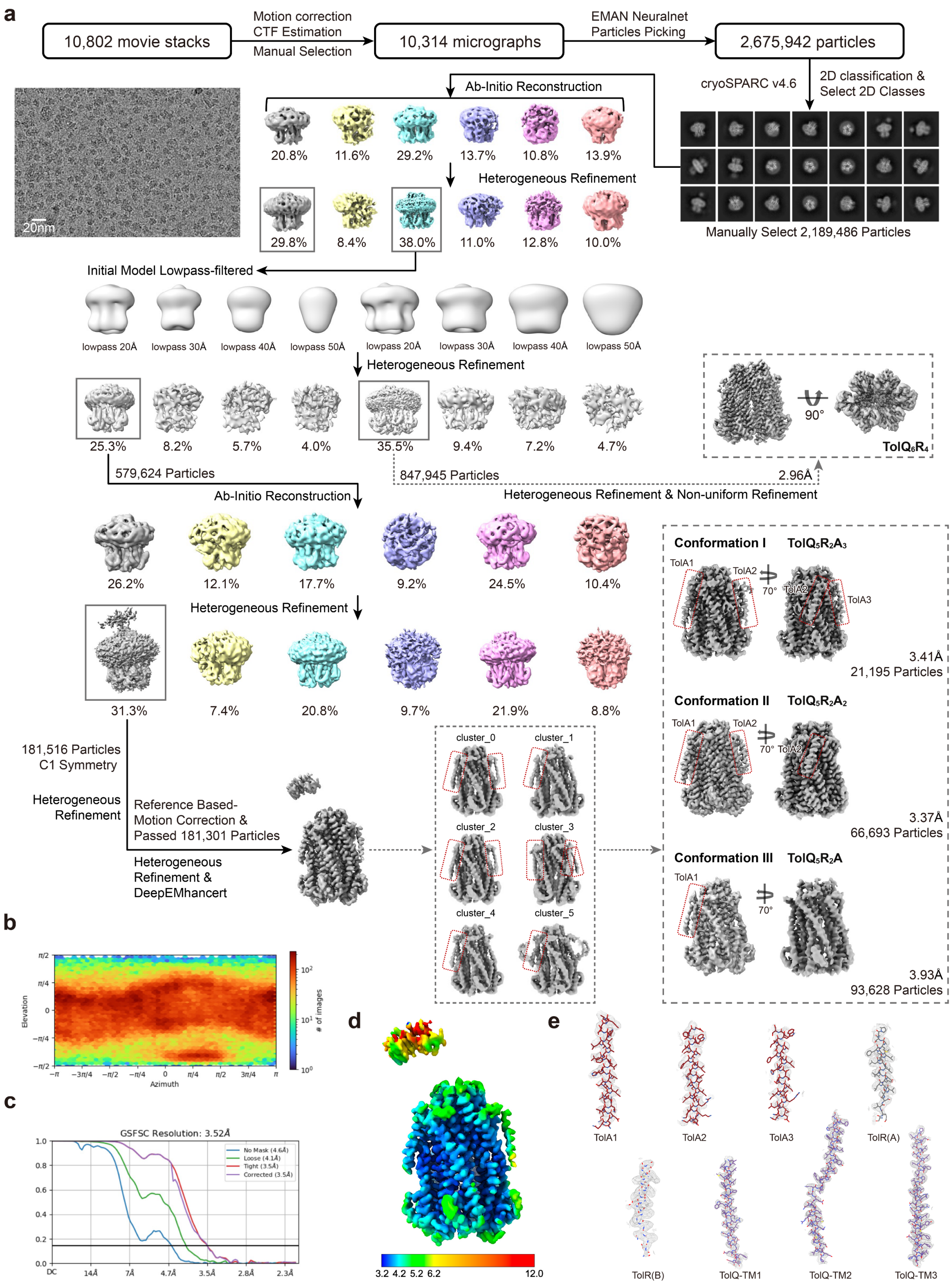

Fig. 2 | Cryo-EM reconstruction of TolQRA structure in nanodiscs.

(a) Workflow of cryo-EM data processing for TolQRA nanodiscs.

(b) Angular distribution histograms of the final reconstruction, as generated by cryoSPARC.

(c) Gold-standard Fourier shell correlation (FSC) curves of the final refined map, showing unmasked (blue), loosely masked (green), tightly masked (red) and corrected (purple), as computed in cryoSPARC.

(d) Cryo-EM density map colored by local resolution estimation.

(e) Cryo-EM maps fitted with the atom model for the transmembrane helices of TolA, TolR, and TolQ of the nanodiscs $TolQ_5R_2A_3$ structure.

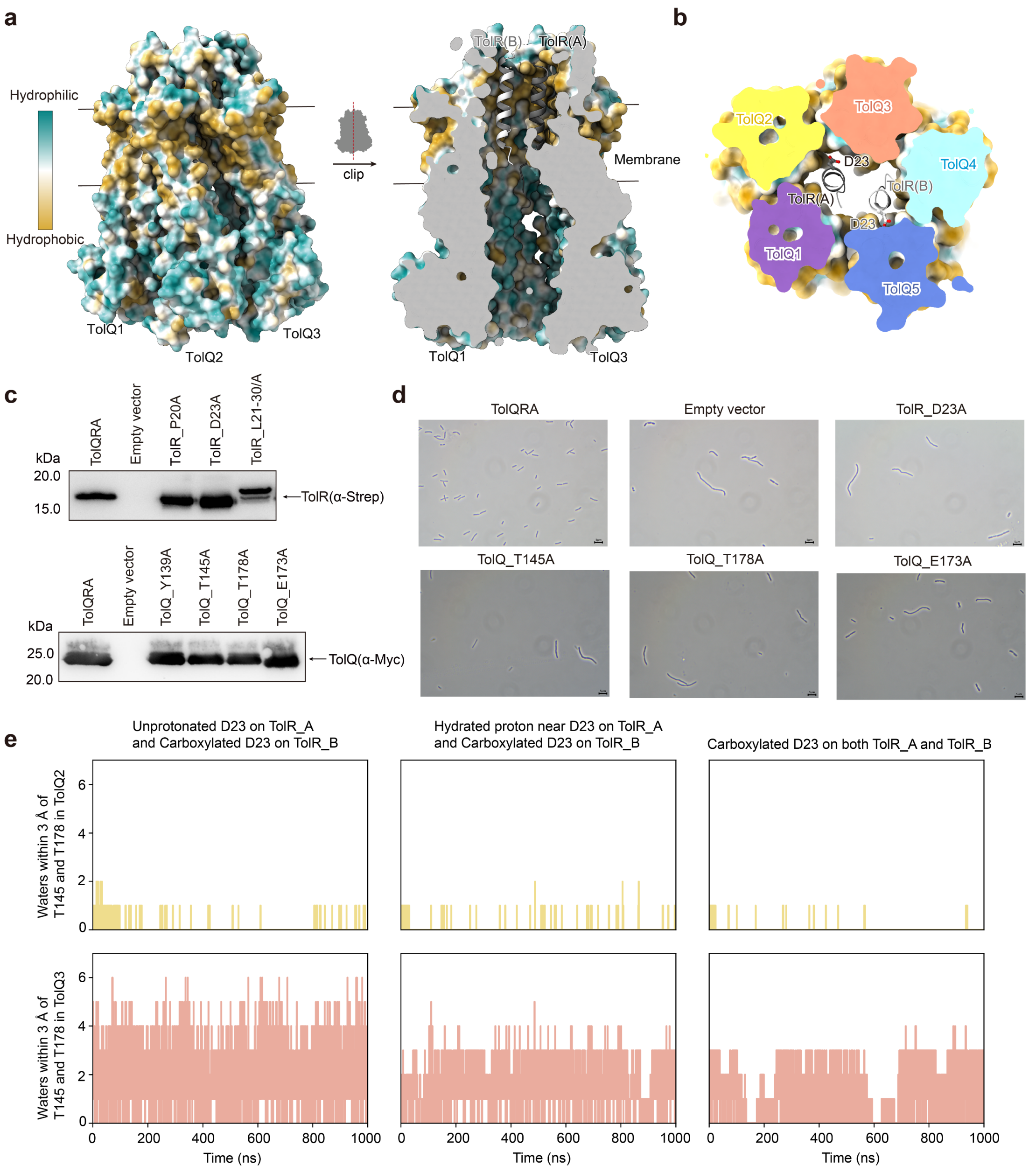

Fig. 3 | Structural characteristics of the TolQRA complex

(a) The surface of the TolQ pentamer illustrates its hydrophobicity distribution (left) and the section showing the cavity enclosing TolR transmembrane helices (right).

(b) The top view of the structure shows that D23 (TolR chain A) directed toward the interface of TolQ2 and TolQ3, while D23 (TolR chain B) points to TolQ5.

(c) The protein expression of mutants corresponds to Figure 2c.

(d) The bacterial morphology of TolQRA wild-type and mutants was captured using phase contrast microscopy. The data in c and d are representative of n = 3 independent experiments.

(e) Molecular dynamics simulations of the water molecules around the T145 and T178 within TolQ2 and TolQ3 subunits.

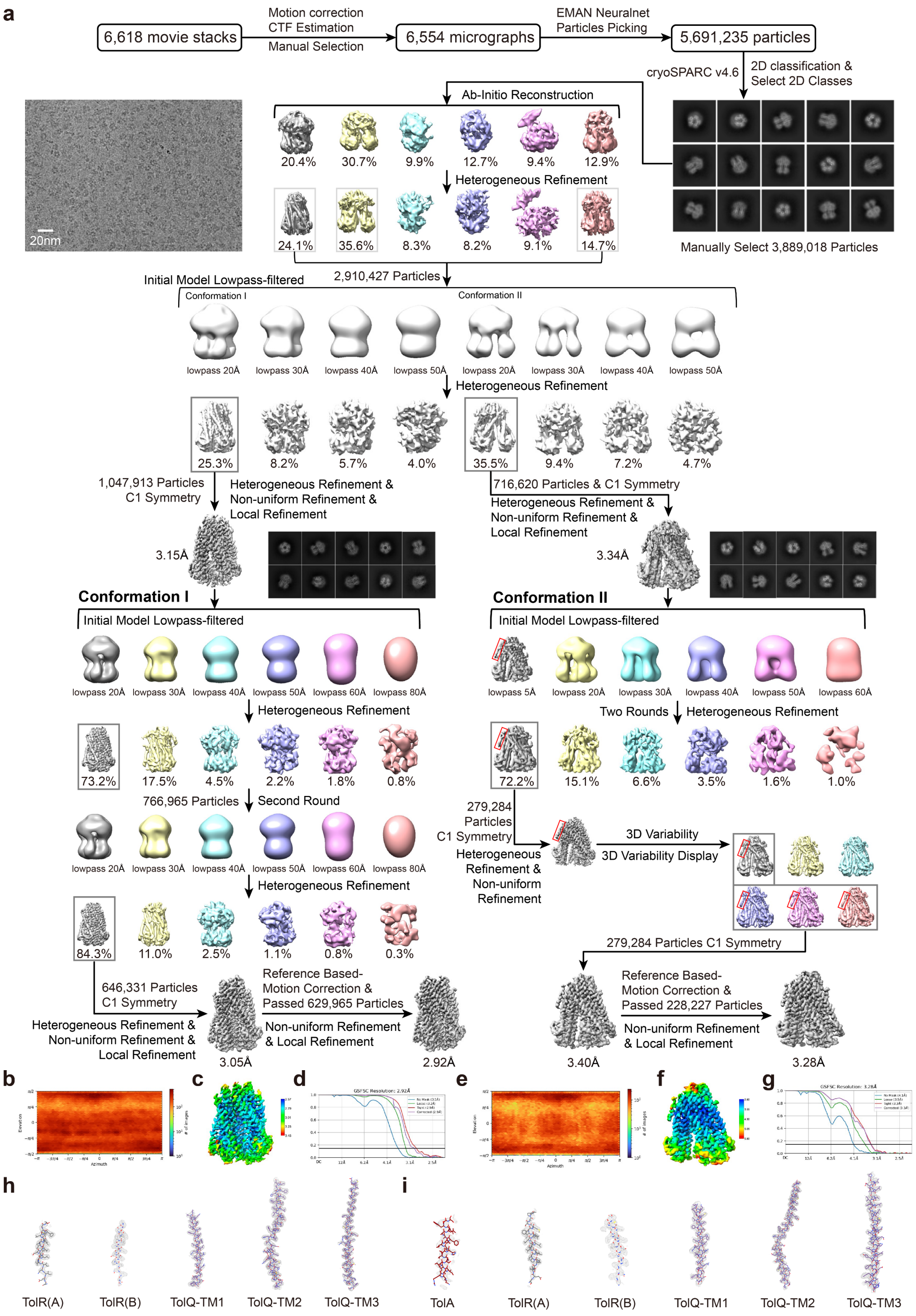

Fig. 4 | Cryo-EM reconstruction of TolQRA reconstituted in SMA

(a) Workflow of cryo-EM data processing for TolQRA SMA.

(b,e) Angular distribution histograms of the final conformation I and conformation II reconstructions, as generated by cryoSPARC.

(c,f) Cryo-EM density maps colored by local resolution estimation.

(d,g) Gold-standard Fourier shell correlation (FSC) curves of the final refined maps,

showing unmasked (blue), loosely masked (green), tightly masked (red) and corrected (purple), as computed in cryoSPARC.

(h,i) Cryo-EM maps fitted with the atom model for the transmembrane helices of TolA, TolR and TolQ of the SMA $TolQ_5R_2$ (h) and $TolQ_5R_2A$ (i) structures.

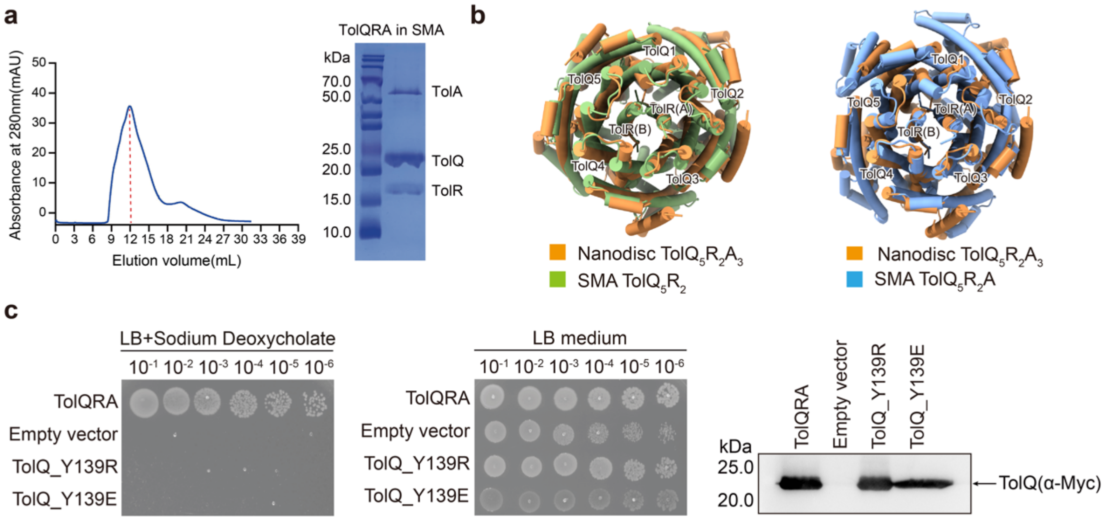

Fig. 5 | Structural comparison of $TolQ_5R_2A_3$ with $TolQ_5R_2A$ and $TolQ_5R_2$

(a) SDS-PAGE and SEC profile of purified TolQRA using native nanodiscs SMA. SDS-PAGE samples are eluted at the volume indicated by red dotted lines.

(b) Periplasmic view of the superimposed Nanodisc $TolQ_5R_2A_3$ and SMA structures $TolQ_5R_2$ (left) and $TolQ_5R_2A$ (right).

(c) Cell viability assays of mutants of Y139 of TolQ with and without surfactant sodium deoxycholate (left and middle panel). Detection of the TolQ mutant protein corresponding to viability assay (right panel). Data are representative of n = 3 independent experiments.

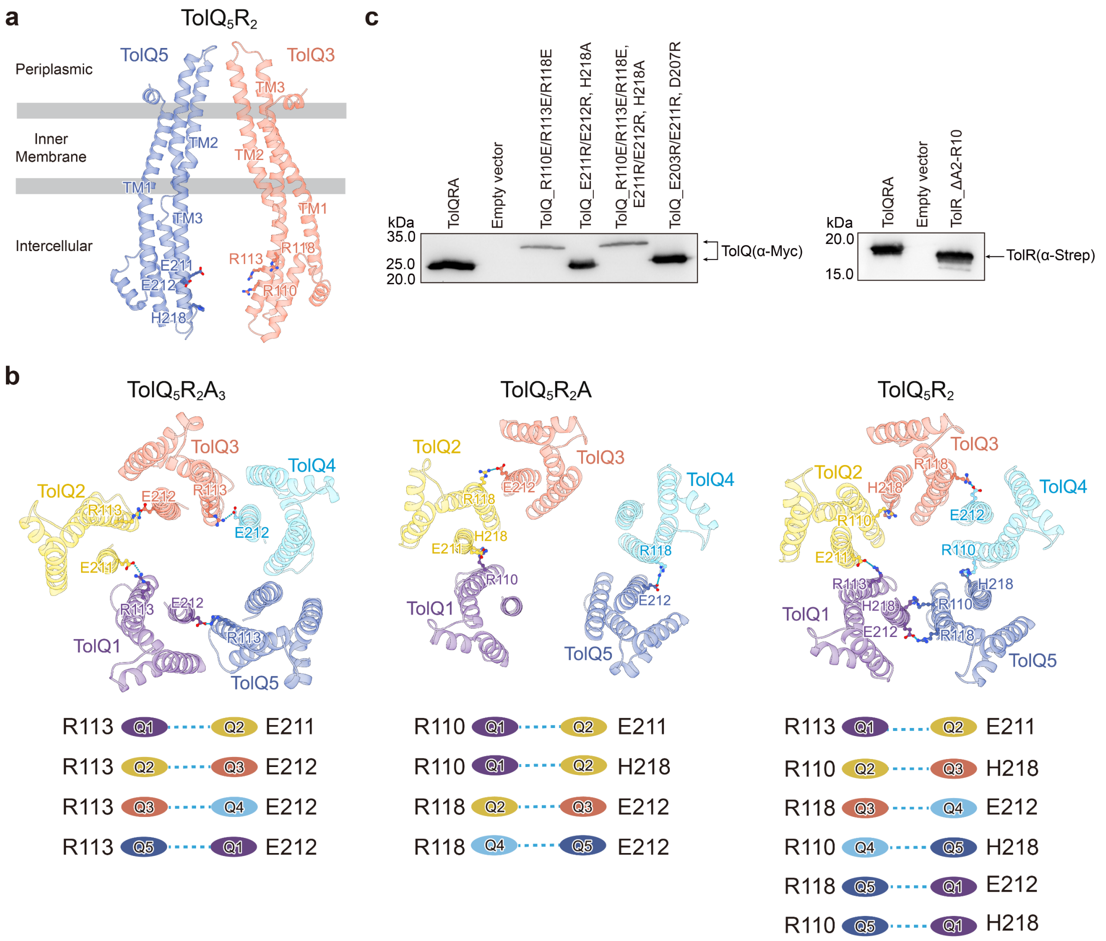

Fig. 6 | Interactions between TolQ subunits in the structure of $TolQ_5R_2A_3$, $TolQ_5R_2A$ and $TolQ_5R_2$

(a) The side view of TolQ5 and TolQ3 highlights the cytoplasmic residues of TM2 and TM3 involved in oligomer stabilization, as shown in (b).

(b) The top view shows the salt bridges and hydrogen bonds formed between the TolQ subunits. The side chains of the interactive residues for each TolQ subunit are labeled: $TolQ_5R_2A_3$ on the left, $TolQ_5R_2A$ in the middle, and $TolQ_5R_2$ on the right. The lower panel summarizes the interacting amino acids.

(c) Detection of the TolQ and TolR mutant proteins corresponding to Figure 3d. Data are representative of n = 3 independent experiments.

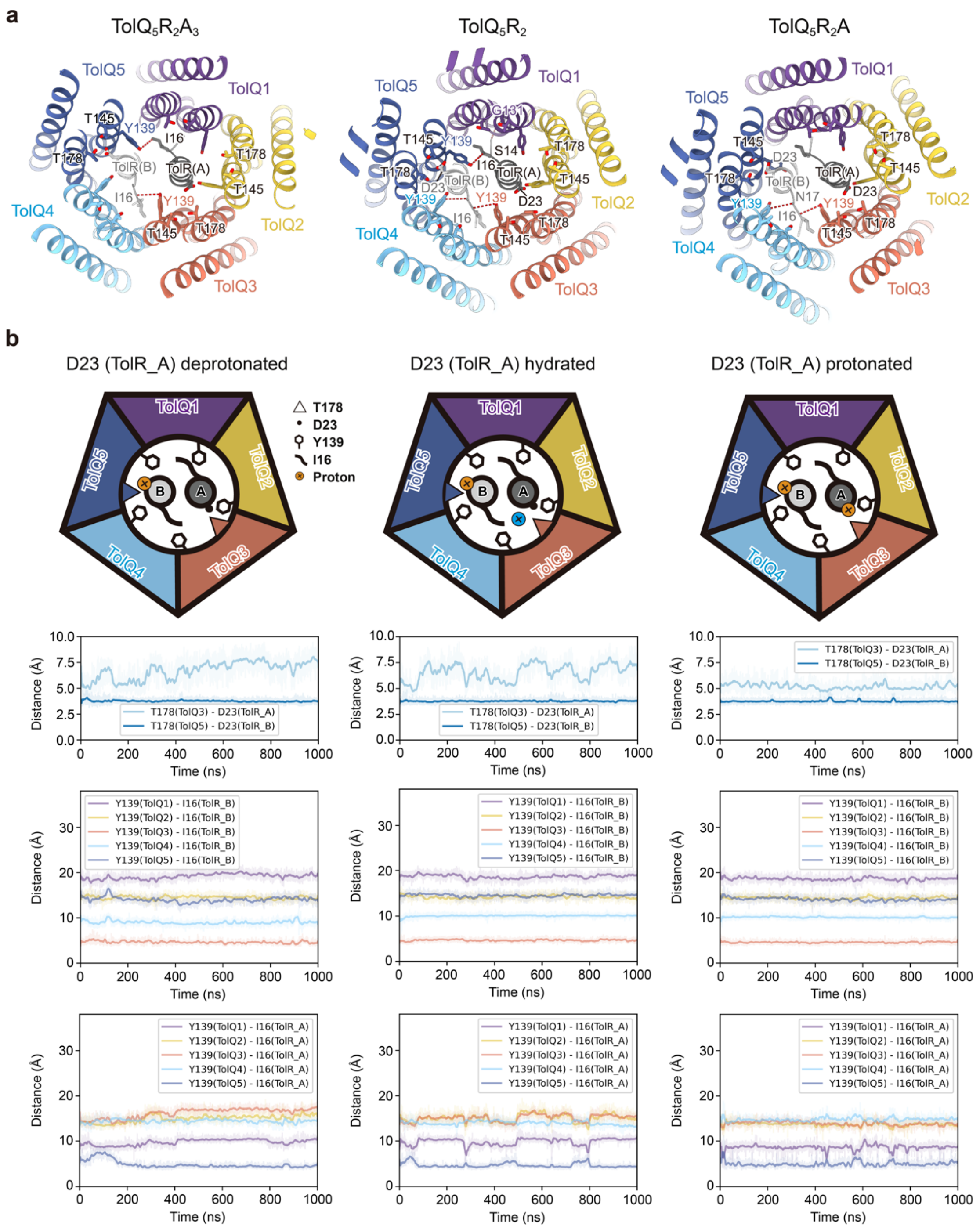

Fig. 7 | Rotary dynamic analysis of the motor unit

(a) Top viewed cavity of the TolQ$_5$R$_2$A$_3$, TolQ$_5$R$_2$, and TolQ$_5$R$_2$A structures from left to right, showing the interactions between the N-terminal residue of TolR dimer and TolQ subunits.

(b) Schematic diagram of a proton-driven TolQ rotation around TolR (upper panel), correlating to the simulated states below. The MD simulation of the SMA TolQ$_5$R$_2$ structure under distinct protonation states (lower panel), measuring the distance fluctuation between TolQ_T178 and TolR_D23, and TolQ_Y139 and TolR_I16.

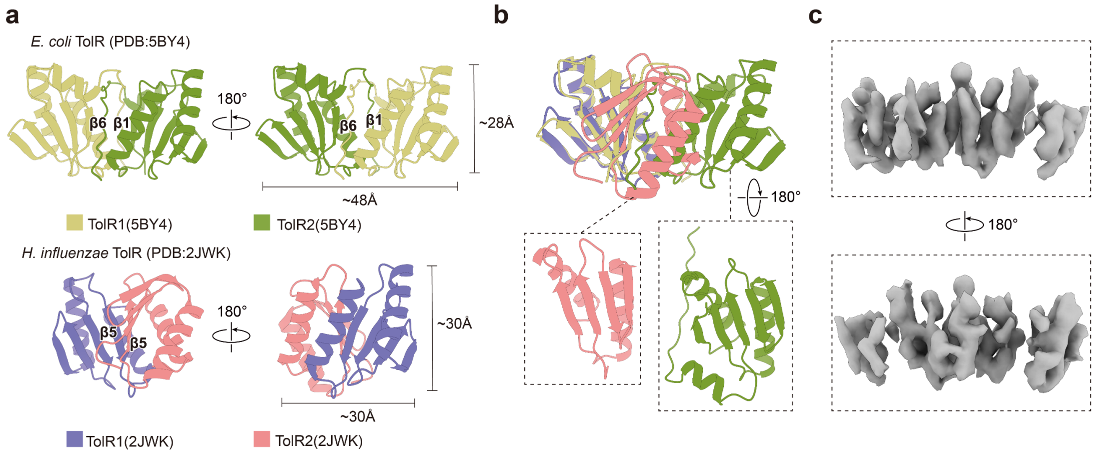

Fig. 8 | Characteristics of the periplasmic domain of TolQRA

(a) The structures of the periplasmic domains of *E. coli* TolR and *H. influenzae* TolR are presented in two states: a front view on the left and a back view on the right. In *E. coli* TolR, the two subunits are colored dark khaki and lime green, respectively. In *H. influenzae* TolR, the two subunits are colored medium blue and hot pink, respectively. The length and width of the entire TolR dimer are indicated. The β-strands that comprise the dimerization interface are labeled.

(b) Superposition of the structures of *E. coli* TolR and *H. influenzae* TolR shows that *E. coli* TolR chain B rotates 180 degrees, indicating a conformational change similar to that in *H. influenzae* TolR.

(c) The front and back views of the periplasmic density in our TolQRA structure.

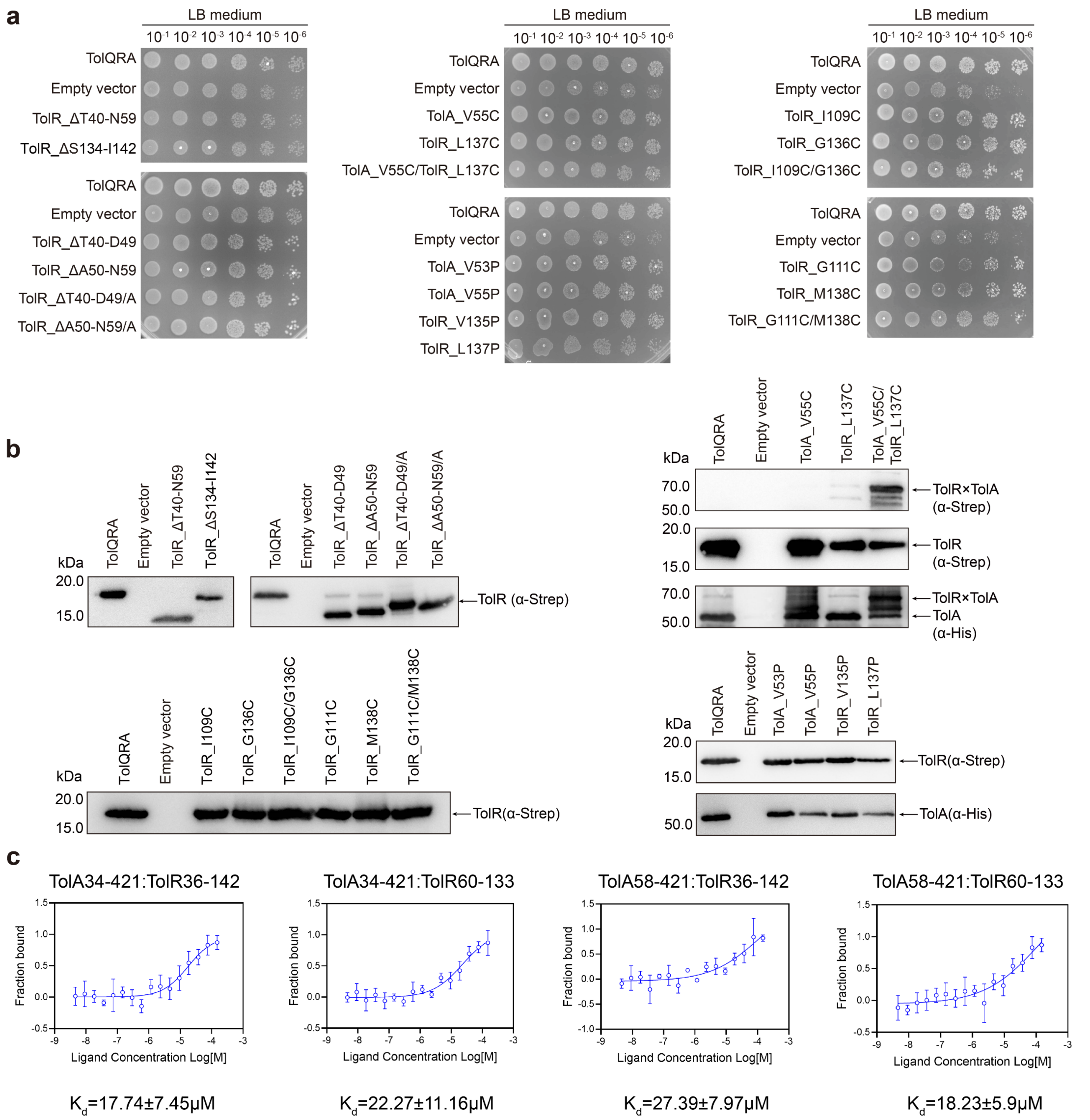

Fig. 9 | Assessments of the interactions between the periplasmic domains of TolR and TolA

(a) Viabilities of the mutants shown in Figure 4g-i without growth pressure.

(b) Western blots show the expression levels of the TolR (left panel, corresponding to Fig. 4g, i) and TolA (right panel, bottom, corresponding to Fig. 4h) mutants, as well as the successful crosslinking of the TolR_β6-TolA_β complex through engineered cysteine residues (right panel, top).

(c) Microscale thermophoresis (MST) analysis of the binding between full-length TolA (TolA34-421) or truncated TolA (TolA58-421) and TolR periplasmic fragments (TolR36-142 or TolR60-133). Error bars indicate the standard deviation from n = 3 independent experimental replicates. Data are presented as mean ± SD.

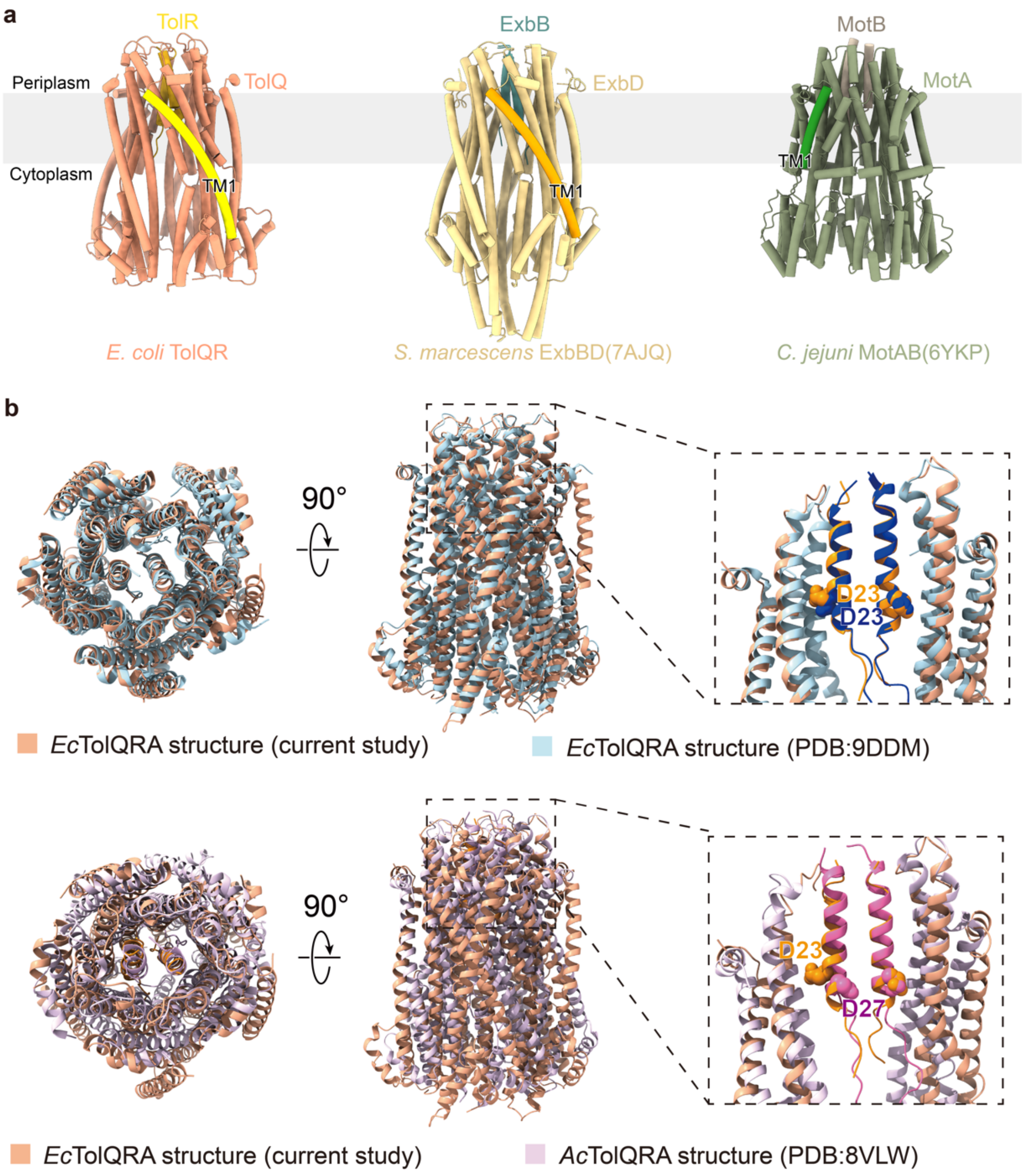

Fig. 10 | Structural comparisons of the related structures of TolQRA, ExbBD, and MotAB

(a) Our TolQR structure is compared to the reported ExbBD (PDB: 7AJQ) and MotAB (PDB: 6YKP) structures.TM1 helices in these structures are highlighted. TolQ is displayed in salmon, TM1 in bright yellow, and TolR in goldenrod. ExbD is presented in pale yellow, TM1 in orange, and ExbD in cadet blue. MotA is illustrated in sea green, TM1 in lime, and MotB in tan.

(b) Superimpositions of our *E. coli* TolQ$_5$R$_2$A$_3$ with recently reported *E. coli* and *Acinetobacter baumannii* TolQRA structures (PDB: 9DDM and 8VLW, respectively), showing conformational differences in TolQ and TolR.

Supplementary Table 1. Cryo-EM data collection, refinement, and validation statistics.

	TolQRA nanodisc (PDB-9QVD, EMD-53394)	TolQRA SMA	
		Conformation I (PDB-9O40, EMD-70088)	Conformation II (PDB-9QUQ, EMD-53380)
**Data collection and processing**			
**Magnification**	130,000×		
**Voltage (kV)**	300		
**Electron exposure ($e^-/Å^2$)**	57.6	45.7	
**Defocus range (μm)**	0.3-2.7	0.6-2.5	
**Pixel size (Å)**	1.1		
**Symmetry imposed**	C1		
**Initial particle images (no.)**	579,624	1,047,913	716,620
**Final particle images (no.)**	181,301	629,965	228,227
**Map resolution (Å)**<br>**FSC threshold**	3.52<br>0.143	2.92<br>0.143	3.28<br>0.143
**Map resolution range (Å)**	3.2～6.2	2.5~3.4	2.8~5.8
**Initial model used (PDB code)**	Alphafold2-predicted model	Alphafold2-predicted model	Alphafold2-predicted model
**Model resolution (Å)**<br>**FSC threshold**	2.80<br>0.143	2.10<br>0.143	2.10<br>0.143
**Map sharpening *B* factor ($Å^2$)**	-113	-142.2	-126.3
**Non-hydrogen atoms**<br>**Protein residues**<br>**Ligands**	9570<br>1220<br>0	8967<br>1143<br>0	9178<br>1170<br>0
**Protein**<br>**Ligand**	87.28<br>0	61.87<br>0	86.53<br>0
**Bond lengths (Å)**<br>**Bond angles (°)**	0.002<br>0.437	0.003<br>0.472	0.003<br>0.435
**Refined model CC**<br>**MolProbity score**<br>**Clashscore**<br>**Poor rotamers (%)**	0.77<br>1.03<br>2.47<br>0.00	0.79<br>1.08<br>2.37<br>0.00	0.75<br>1.10<br>3.07<br>0.00
**Favored (%)**<br>**Allowed (%)**<br>**Disallowed (%)**	98.42<br>1.58<br>0.00	97.70<br>2.30<br>0.00	98.53<br>1.47<br>0.00
